# Synergistic Effect of Guanidinium and Tertiary Amine Groups on Boosting Gene Delivery

**DOI:** 10.1021/jacsau.5c00592

**Published:** 2025-07-23

**Authors:** Yinghao Li, Jiahao Liu, Liang Yao, Tianyu Mao, Xinyue Wang, Zhonglei He, Jing Lyu, Wenxin Wang

**Affiliations:** † Institute of Precision Medicine (AUST-IPM), 91594 Anhui University of Science and Technology, Huainan, China; ‡ Charles Institute of Dermatology, School of Medicine, 8797 University College Dublin, Dublin 4, Ireland; § Centre of Micro/Nano Manufacturing Technology (MNMT-Dublin), School of Mechanical & Materials Engineering, 8797 University College Dublin, Dublin 4, Ireland

**Keywords:** nucleic acid delivery, poly(β-amino ester), polymeric vector, synergetic effect, guanidinium

## Abstract

The lack of safe and efficient delivery vectors remains a major bottleneck in nucleic acid therapeutics, limiting clinical translation. In this study, by incorporating the synergistic effects of guanidinium and tertiary amine functional groups, a new versatile branched poly­(β-amino ester) (PAE)-based delivery system was designed and developed, resulting in enhanced nucleic acid (DNA/mRNA) delivery. The synergistic interactions between these functional groups were systematically analyzed using 2D-NMR, AFM, TEM, and photophysical characterization. The results show that self-assembly occurs when polymers containing different functional groups are mixed, which changes the interactions between molecules and significantly affects the structure of the polyplex. This synergy was found to significantly enhance cellular uptake, contributing to improved transfection efficiency. Compared to the leading commercial reagents (Lipofectamine 3000, jetPEI, Xfect, and Lipofectamine MessengerMAX), the optimized polymeric vector formulations demonstrated superior transfection efficiency *in vitro* with both DNA and mRNA. Their high transfection performance was also confirmed *in vivo*, along with reduced cytotoxicity and tunable organ-targeting property.

## Graphical abstract

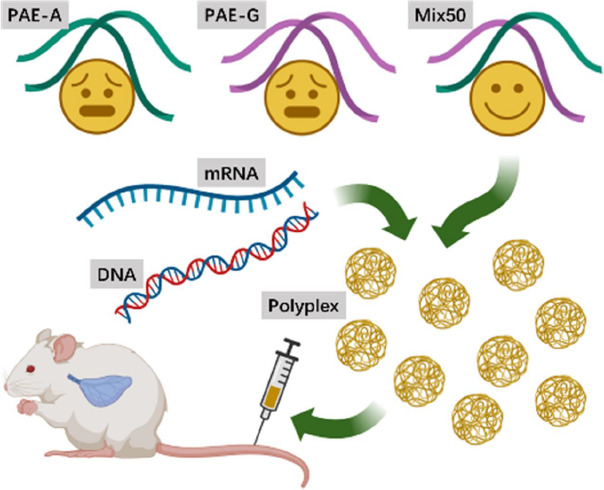

## Introduction

Nucleic acid therapy systems, particularly those for DNA and mRNA, are at the forefront of biomedical research due to their transformative potential in precision medicine. Nucleic acid drugs enable precise and efficient gene regulation, offering promising therapeutic options for genetic disorders, infectious diseases, and cancer.^1–5^ During the COVID-19 pandemic, the rapid global deployment of mRNA vaccines by Pfizer-BioNTech and Moderna highlighted the potential of nucleic acid delivery systems to address urgent healthcare challenges.^6–8^ These vaccines, encoding the viral spike protein, triggered robust immune responses, demonstrating safety and adaptability to viral mutations. Consequently, nucleic acid delivery has become a foundation of next-generation therapies, driving advancements in precision medicine and fostering a rapidly growing international market.

Efficient delivery vectors are critical to advancing the therapeutic potential of nucleic acids. Current mainstream vectors include inactivated viruses, liposomes, and polymers. Among these, polymer-based systems represent an important development direction.^9–11^ Compared to viral vectors, polymers offer higher gene-loading capacity,^12,13^ and they exhibit better stability and modifiability compared to liposomes.^14–16^ After decades of research, a range of polymer vectors such as polyethylenimine (PEI, Polyplus- Sartorius) and poly­(β-amino ester)­s (PAEs, Xfect- Takara Bio Inc.) have reached the market. However, challenges such as transfection efficiency, cellular uptake, and biocompatibility still remain. Therefore, continued efforts are being devoted to the development of next-generation polymer vectors with improved performance.

Delivering nucleic acids involves overcoming multiple barriers during complex *in vitro* or *in vivo* processes.^17–19^ Successful and efficient transfection requires a delicate balance of multiple functionalities within the vector. Consequently, the development of polymer vectors has grown increasingly sophisticated. Compared to early single-component polymers, scientists are now incorporating multiple functional groups into a single polymer or combining multiple components with distinct roles.^9–11,9–11,20–22^ Among the various functional groups explored, guanidinium and amine groups have been widely studied and recognized.^23–26^ Guanidinium groups have been extensively reported for their ability to enhance the cellular uptake of nanoparticles and boost the proton sponge effect, while amine groups, due to their protonation ability which imparts a positive charge to the polymer and facilitates binding with negatively charged DNA, as well as aiding endosomal escape, are indispensable components of nearly all polymer vectors. Despite their individual advantages, the potential interactions between these groups and their synergetic effects on delivery and transfection remain underexplored. This gap raises the possibility that their combination may lead to unique or enhanced properties.

Therefore, in this study, a series of branched PAEs that possess guanidinium, tertiary amine, and both functional groups (at different ratios) were designed and prepared, respectively. Their structures, topologies, and the interactions between guanidinium and tertiary amine groups were systematically characterized using gel permeation chromatography (GPC), nuclear magnetic resonance (NMR) (^1^H-, ^13^C-, and 2D-NMR), atomic force microscopy (AFM), transmission electron microscopy (TEM), Zetasizer, and photophysical analysis. These new PAE vectors were applied to DNA and mRNA delivery. It was observed that when PAEs bearing two different functional groups are mixed, self-assembly occurs, thereby altering the intermolecular interactions. This synergetic effect ultimately results in modifications to the polyplex structure, an increase in the cellular uptake efficiency of the polyplexes, and a significant improvement in gene delivery performance.

## Results and Discussion

### Synthesis and Characterization of PAEs with Different Functional Groups

Based on previous work,^27–29^ the monomer combination of 1,4-butanediol diacrylate (BDA), pentaerythritol tetraacrylate (PTTA), and 5-amino-1-pentanol (AP) was used to construct the PAE backbone (where PTTA serves as a branching monomer). Through a simple Michael addition reaction (Scheme 1), the acrylate monomers BDA and PTTA reacted directly with the amine monomer AP. 1-(3-aminopropyl)-4-methylpiperazine (E7) was employed for end-capping, generating a PAE with terminal tertiary amine groups (named as PAE-A in the following text, Figures S1 to S5). The guanidinium-terminated polymer (PAE-G) was obtained by a post chemical modification process of PAE-A using 4-guanidinobenzoic acid hydrochloride (GAH) (Scheme S1, Figures S2, S3, S4 and S6). The detailed synthetic procedures were provided in Supporting Information.

**Scheme 1:**
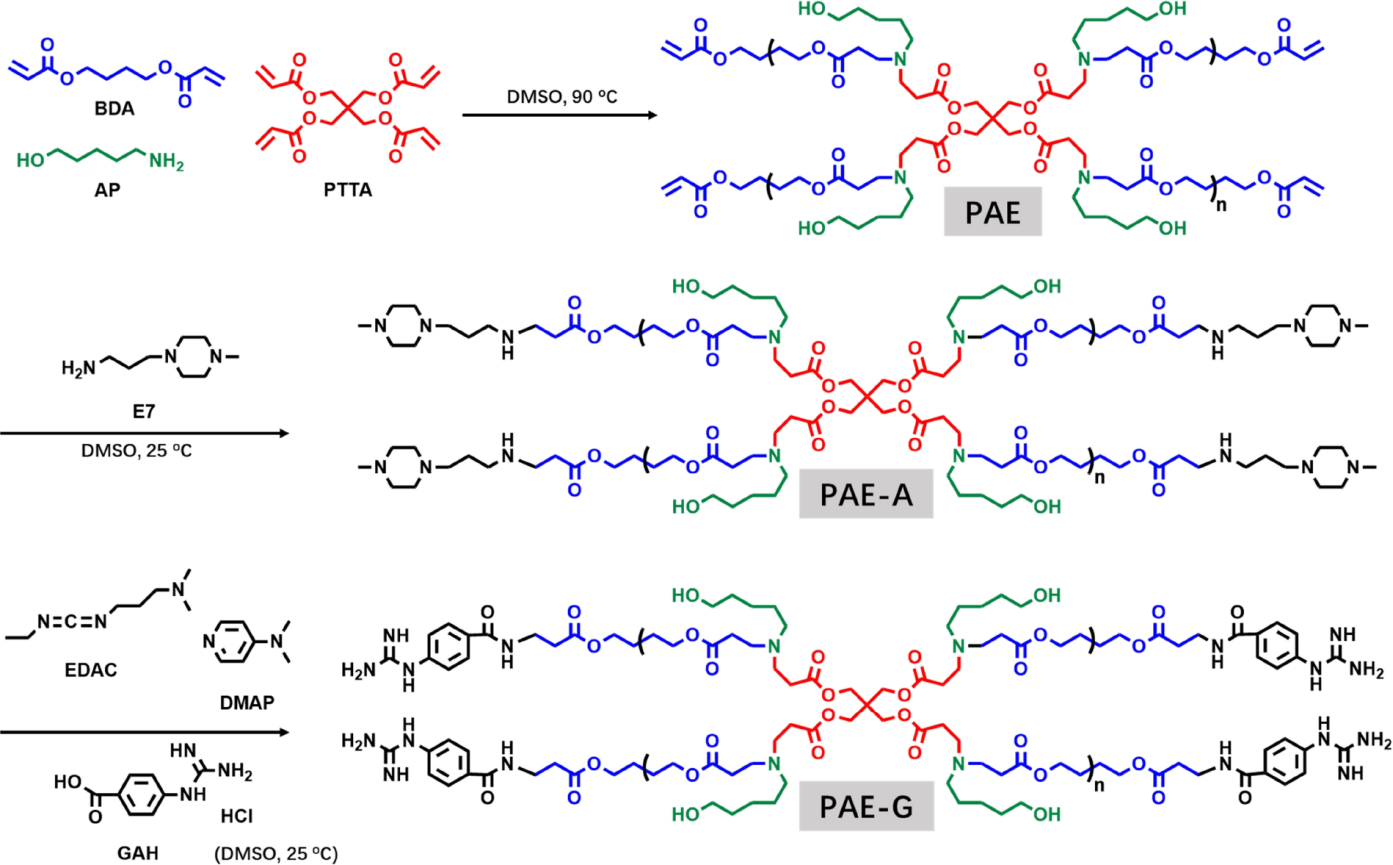
Schematic Diagram of the Synthetic Routes for PAE-A and PAE-G

Subsequently, the synthesized PAE-A and PAE-G were characterized by GPC and NMR to confirm their structures. GPC analysis revealed that the weight-average molecular weight (*M*_w,GPC_) of PAE-A was approximately 28 kDa (Figure 1A). However, after guanidinium modification, no GPC signal was detected due to the strong affinity of guanidinium groups for the column packing material. Therefore, NMR analysis was adopted to confirm the structure (Figures S2, S3, S4). It can be seen that compared to PAE-A, the peak shapes and positions corresponding to AP (^1^H NMR: f, d, e, Figure S2) in PAE-G remained unchanged, suggesting no condensation reactions involving AP occurred during PAE-G synthesis. Additionally, the peaks corresponding to BDA (b, g, Figures S2, S3) exhibited no shifts or changes, indicating that the polymer backbone was not degraded. While, as expected, the peaks corresponding to E7 disappeared (a, Figure S2. S3), which were replaced by the peaks of guanidinium (p, q, r, s, t, Figure S2). Similar changes were also observed in the ^13^C NMR spectrum (Figure S4). These results prove that PAE-A and PAE-G were successfully synthesized, which share an identical backbone, differing only in their terminal groups, which are guanidinium and tertiary amine groups, respectively.

**Figure 1:**
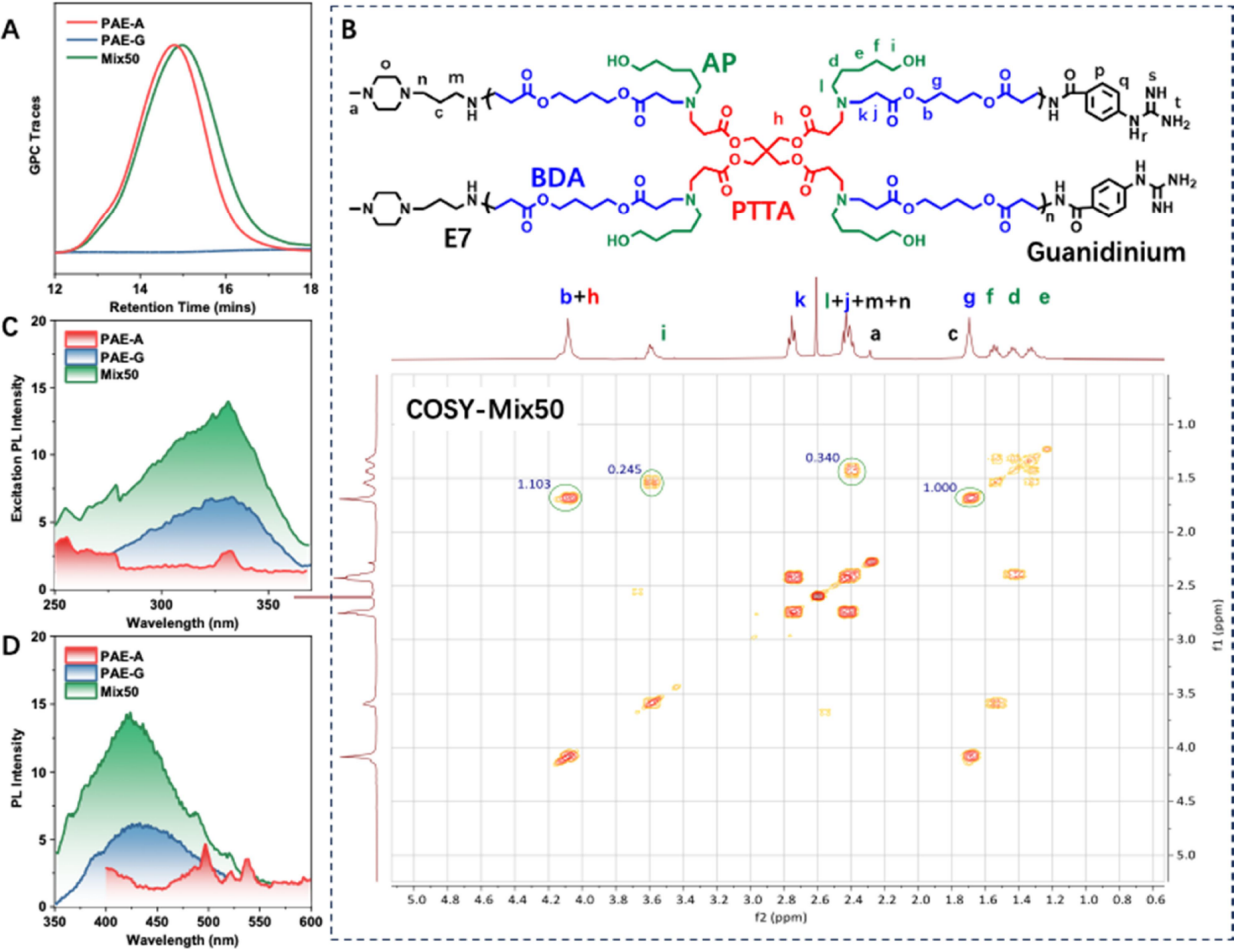
Characterization of polymer structures and interactions. (A) GPC traces of PAE-A, PAE-G, and PAE-Mix50. (B) ^1^H, ^1^H–COSY spectrum of PAE-Mix50. Schematic representation of the chemical structures of PAE-A, PAE-G, and PAE-Mix50 only for indicating the characteristic functional groups on different polymers. (C) Excitation spectra of the polymers at 425 nm. (D) Photoluminescence (PL) spectra of the polymers. Measurements were conducted by dissolving 10 μL of a 100 mg/mL polymer stock solution in 3 mL pure water.

Based on the confirmed structure, the third polymer – PAE-Mix50 – was prepared by directly mixing PAE-A and PAE-G in a 1:1 ratio. GPC analysis (Figure 1A) showed that the elution peak of PAE-Mix50 shifted significantly to the right, and the *M*_w,GPC_ decreased to 23 kDa. Considering that no degradation was observed in the NMR spectra (Figures S2, S3, S4), this shifted GPC trace preliminarily suggested an interaction between the guanidinium groups and the amine groups in PAE-Mix50 - due to the inability of PAE-G to pass through the GPC column normally, it exerted a ″dragging″ effect on PAE-A, altering PAE-Mix50s elution profile. To further investigate this interaction, 2D-NMR characterization was conducted (Table S1). The short-range ^1^H–^1^H correlation spectra (COSY) (Figure 1B, Figures S5 and S6), and the ^1^H–^1^H total correlation spectra (TOCSY) (Figures S7 to S9) revealed that AP became more extended after mixing (in PAE-Mix50, the coupling peaks of i and f decreased compared to the individual PAE-A and PAE-G). Simultaneously, the coupling peaks of n and o in PAE-A disappeared after mixing. These observations indicate that some originally coiled structures became more extended upon mixing.

Since neither PAE-A nor PAE-G possesses a conjugated backbone, their photoluminescent (PL) behavior arises from the aggregation of lone-pair-containing functional groups in spatial proximity. Thereby, the interactions of different functional groups in PAE-Mix50 can be reflected by the photophysical properties of three polymers. As shown in Figure S10, the luminescence intensity of all three polymers increased with concentration. However, at the same concentration, PAE-A exhibited relatively weak luminescence in both the excitation spectrum (Figure 1C) and the emission spectrum (Figure 1D), while PAE-G showed a weak emission peak at around 430 nm. Notably, in PAE-Mix50, the luminescence was significantly enhanced in both spectra, which provided further evidence for the synergetic interaction between PAE-A and PAE-G within PAE-Mix50, leading to the local aggregation of functional groups.

To more intuitively observe the interactions between the polymers, PAE-A, PAE-G, and PAE-Mix50 were characterized using AFM (Figure 2A). It is found that PAE-A, being too soft and exhibiting weak adhesion to mica, could not be properly characterized - during probe tapping, it was pushed away, leaving traces resembling glacial erosion (Figure 2 **A (i)**). To stabilize its surface morphology, the PAE-A sample was coated with titanium using a PVD process. For PAE-G, large particles on the scale of tens of microns were observed (Figure 2 **A (iii)**). Compared to the soft, droplet-like collapse of PAE-A on the substrate (Figure 2 **A (ii)**), PAE-G formed more compact and three-dimensional structures resembling gravel. In contrast, PAE-Mix50 exhibited entirely different morphologies. Two distinct structures were captured: one resembled irregular droplets with sizes close to 1 μm (Figure 2 **A (iv)**), while the other appeared sea-urchin-like (Figure 2 **A (v) and (vi)**), featuring a dense core with numerous radiating spines, surrounded by structures similar to PAE-G. These unique morphologies of PAE-Mix50 further confirmed the existence of interactions between PAE-A and PAE-G, arising from the cation-π interaction, π-π staking and dipole–dipole interactions (Figure 2B). Further, turbidity measurements showed the good water solubility of all three polymers (Figure S11). DLS characterization revealed that the size of PAE-A molecules in aqueous solution was slightly larger than that of PAE-G, and the size of PAE-Mix50 was even larger (Figure 2C), consistent with the AFM results.

**Figure 2:**
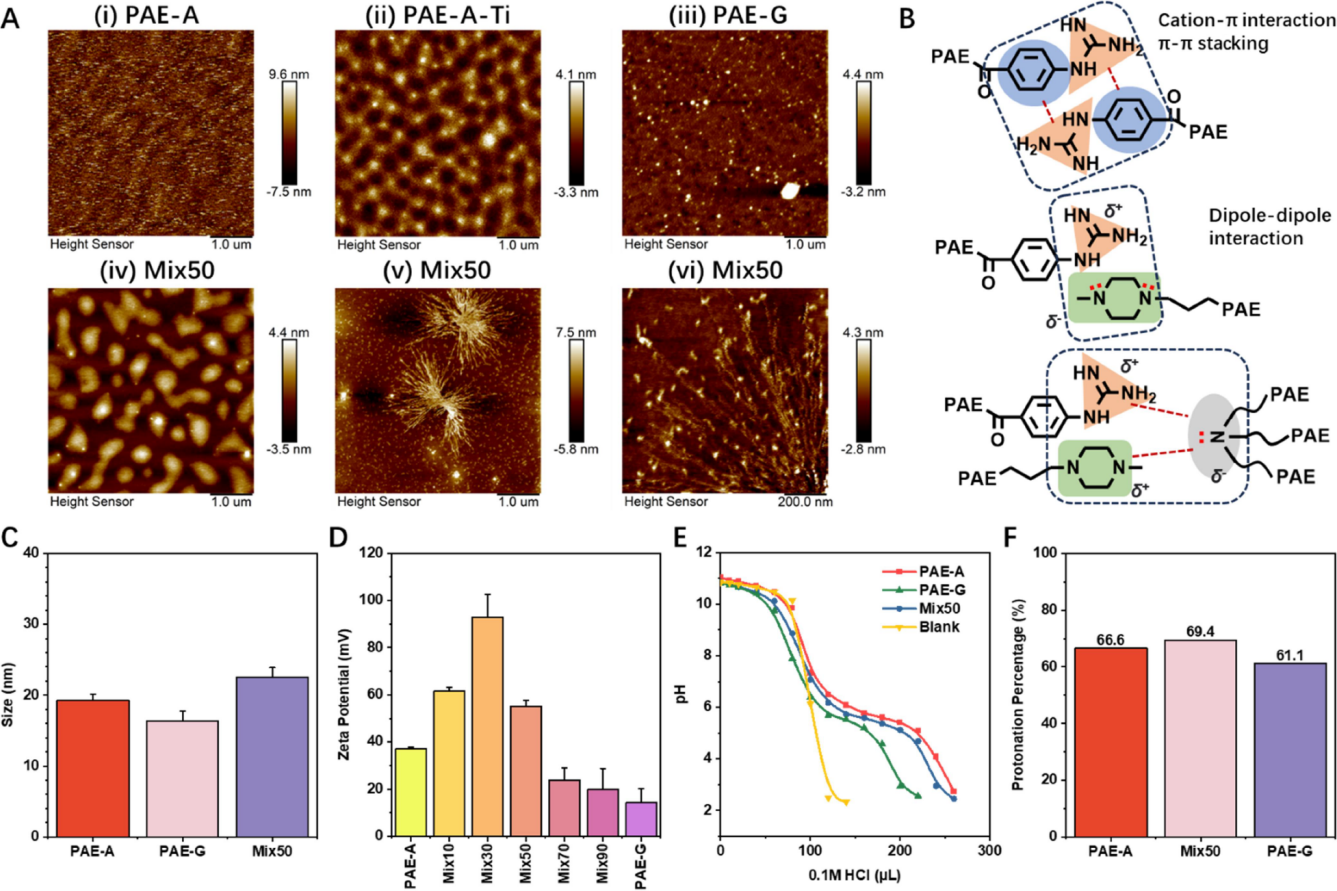
Morphology and properties of polymers in aqueous solution. (A) AFM measured morphology of (i) PAE-A, (ii) PAE-A after PVD fixation, (iii) PAE-G, and (iv)­(v)­(vi) PAE-Mix50 ((vi) is an enlarged view of (v)). The scale bar used is 1 μm. (B) Schematic representation of intermolecular interactions in PAE-Mix50 (from top to bottom: interactions among guanidinium groups, interactions between guanidinium and terminal amine, interactions between guanidinium/terminal amine and backbone amine). (C) Polymer sizes in aqueous solution measured by DLS. (D) Zeta potential of polymers in aqueous solution (PAE-A and PAE-G were mixed in different ratios – 0:100 (PAE-A), 10:90 (PAE-Mix10), 30:70 (PAE-Mix30), 50:50 (PAE-Mix50), 70:30 (PAE-Mix70), 90:10 (PAE-Mix90), and 100:0 (PAE-G)). (E) Acid-titration curves of the polymers. The blank control group consisted of the same amount of DMSO. (F) Protonation levels of different polymers in 25 mM sodium acetate solution.

Additional studies found that after mixing PAE-A and PAE-G in different ratios, the zeta potential of the mixed polymers increased compared to individual PAE-A or PAE-G (Figure 2D), suggesting that the combination of PAE-A and PAE-G altered the charge distribution. The protonation capacity of the mixed materials was then measured. Specifically, acid titration experiments (Figure 2E) showed that although the proton buffering capacity of PAE-G was far lower than that of PAE-A, PAE-Mix50 retained a proton buffering capacity comparable to PAE-A. Besides, the degree of polymer protonation in the acidic sodium acetate buffer was calculated (Figure 2 **F,** Table S2). Among the three polymers, PAE-Mix50 exhibited the highest degree of protonation, reaching 69%, consistent with the trend observed in the zeta potential results. Overall, the above analysis demonstrated the distinct topology of three polymers, and the interactions in PAE-Mix50 made it possess a larger size, higher zeta potential, and the best protonation capability.

### Structure and Properties of Polyplexes Formed by Different PAEs

Next, the combined influence of PAE-A and PAE-G on the structure and properties of polyplexes was examined. To this end, a series of polymers were examined by mixing PAE-A and PAE-G in different ratios (0:100 (PAE-A), 10:90 (PAE-Mix10), 30:70 (PAE-Mix30), 50:50 (PAE-Mix50), 70:30 (PAE-Mix70), 90:10 (PAE-Mix90), and 100:0 (PAE-G)), followed by complexation with DNA in acidic sodium acetate buffer to form various polyplexes. The first property examined was DNA binding efficiency (Figure 3A). The results showed that polyplexes formed with PAE-A alone achieved a binding efficiency of approximately 65%. However, even the addition of just 10% PAE-G increased the DNA binding efficiency to over 90%. Since the protonation levels of the individual polymers differed only slightly (<10%, Figure 2F), the zeta potential of their resulting polyplexes was consistently around +30 mV (Figure 3B). The size of the polyplexes decreased as the proportion of PAE-G in the polymer mixture increased from 10% to 50%, dropping from approximately 200 nm to about 150 nm, and then slightly increased with the further increase of PAE-G proportions (Figure 3C).

**Figure 3:**
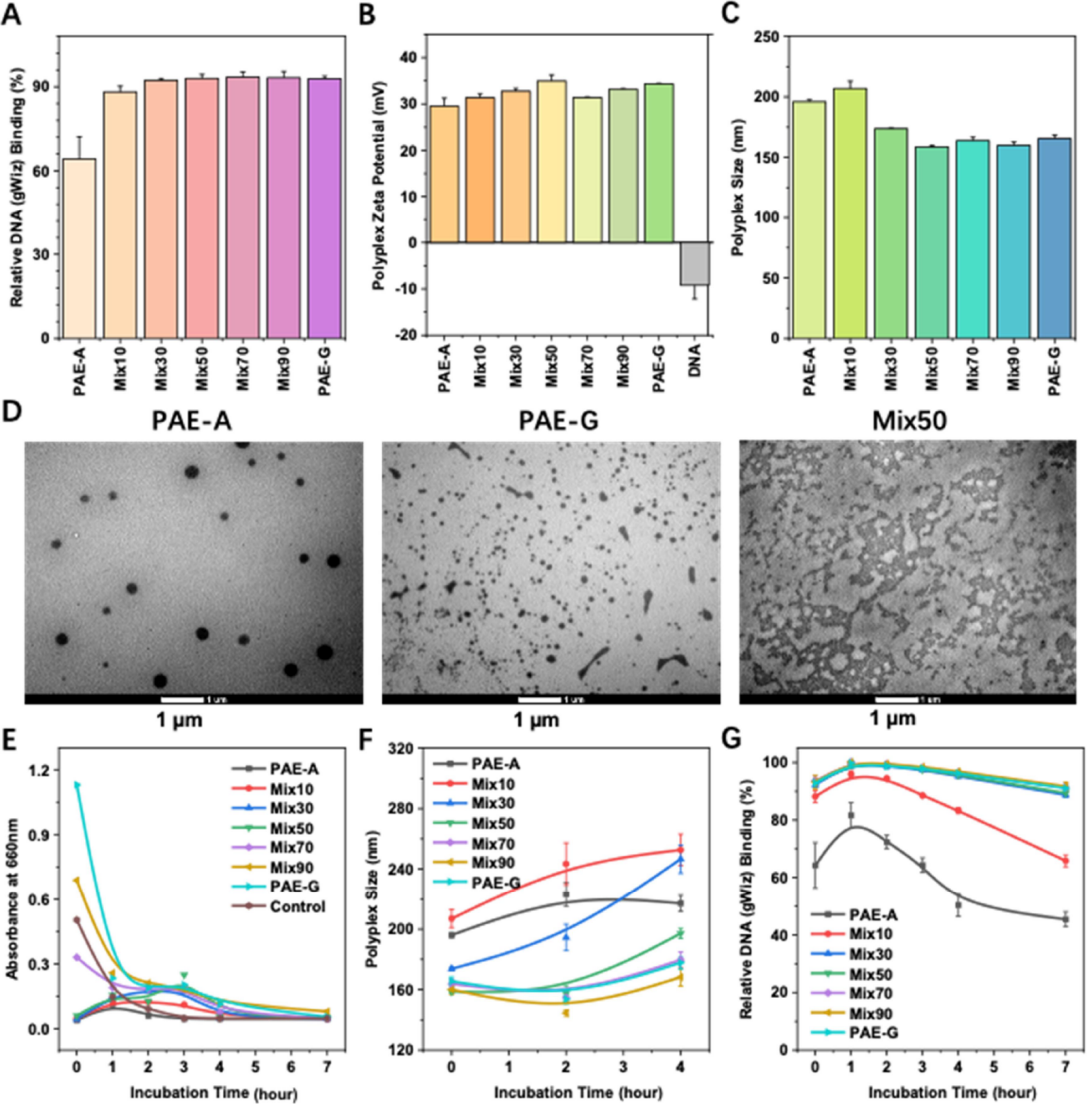
Structural and properties characterization of polymer-DNA polyplexes. (A) DNA binding efficiencies of polyplexes formed with different polymers. (B) Zeta potential of polyplexes formed with different polymers. (C) Size of polyplexes formed with different polymers. (D) TEM images of polyplexes formed with PAE-A, PAE-G, and PAE-Mix50. The scale bar used is 1 μm. (E) Time-dependent stability of polyplexes measured by turbidity. The control group contain no polyplexes. (F) Changes in the size of polyplexes during incubation at 37 °C over time. (G) DNA encapsulation efficiency of polyplexes during incubation at 37 °C over time.

TEM imaging was used to observe the morphology of the polyplexes (Figure 3D). Polyplexes formed solely with PAE-A exhibited dense, spherical structures, whereas those formed with PAE-G alone showed granular or rod-like shapes. In comparison, polyplexes based on PAE-Mix50 displayed irregular, clod-like morphologies. Due to the similar backbone structures of the polymers, darker shadows in the TEM images can indicate higher density.^30^ Therefore, compared to polyplexes formed by individual polymers, it is clear that those formed with PAE-Mix50 exhibited a lower density. This reflects that the synergetic interactions of PAE-A and PAE-G not only altered the morphology of PAE-Mix50 polyplexes but also significantly reduced their packing density.

Finally, the stability of the polyplexes was evaluated. First, the stability of the nanoparticles in 10% serum was investigated using turbidity measurements. As shown in Figure 3 **E,** no significant aggregation was observed for any of the polyplexes (i.e., no obvious increase of the absorbance for all polyplexes), even after 7 h of incubation. Further studies revealed that, except for PAE-Mix30, no significant change was observed from the size (Figure 3F) and zeta potential (Figure S12) of the polyplexes. In addition, DNA binding efficiency was measured at different time points (Figure 3G). Polyplexes formed with PAE-A alone or PAE-Mix10 exhibited relatively weak stability. However, when the PAE-G proportion was increased to 30% or higher, no significant structural changes or DNA release were observed even after 7 h of incubation.

### *In Vitro* Delivery of DNA and mRNA

The above polymers were then applied to DNA delivery *in vitro*. Based on the previous studies, a commercially available plasmid encoding green fluorescent protein (GFP), gWiz, was directly used for delivery experiments in HEK cells. The results in Figure 4A showed that GFP expression initially increased and then decreased as the proportion of PAE-G increased, reaching its peak at PAE-Mix50 (though with a slightly increased cytotoxicity, Figure S13). Flow cytometry was further employed to evaluate transfection efficiency. Interestingly, nearly all polymers achieved close to 100% cellular transfection efficiency (Figures S14, S15). However, GFP expression in cells transfected with PAE-Mix30 to PAE-Mix90 polyplexes significantly exceeded that achieved with PAE-A or PAE-G alone (Figure 4B). More importantly, when compared to commercial transfection reagents, cells transfected with PAE-Mix50 polyplexes expressed GFP at levels surpassing those achieved with leading products such as Lipofectamine 3000, jetPEI, and Xfect (Figure 4 **C,** Figures S16, S17).

**Figure 4:**
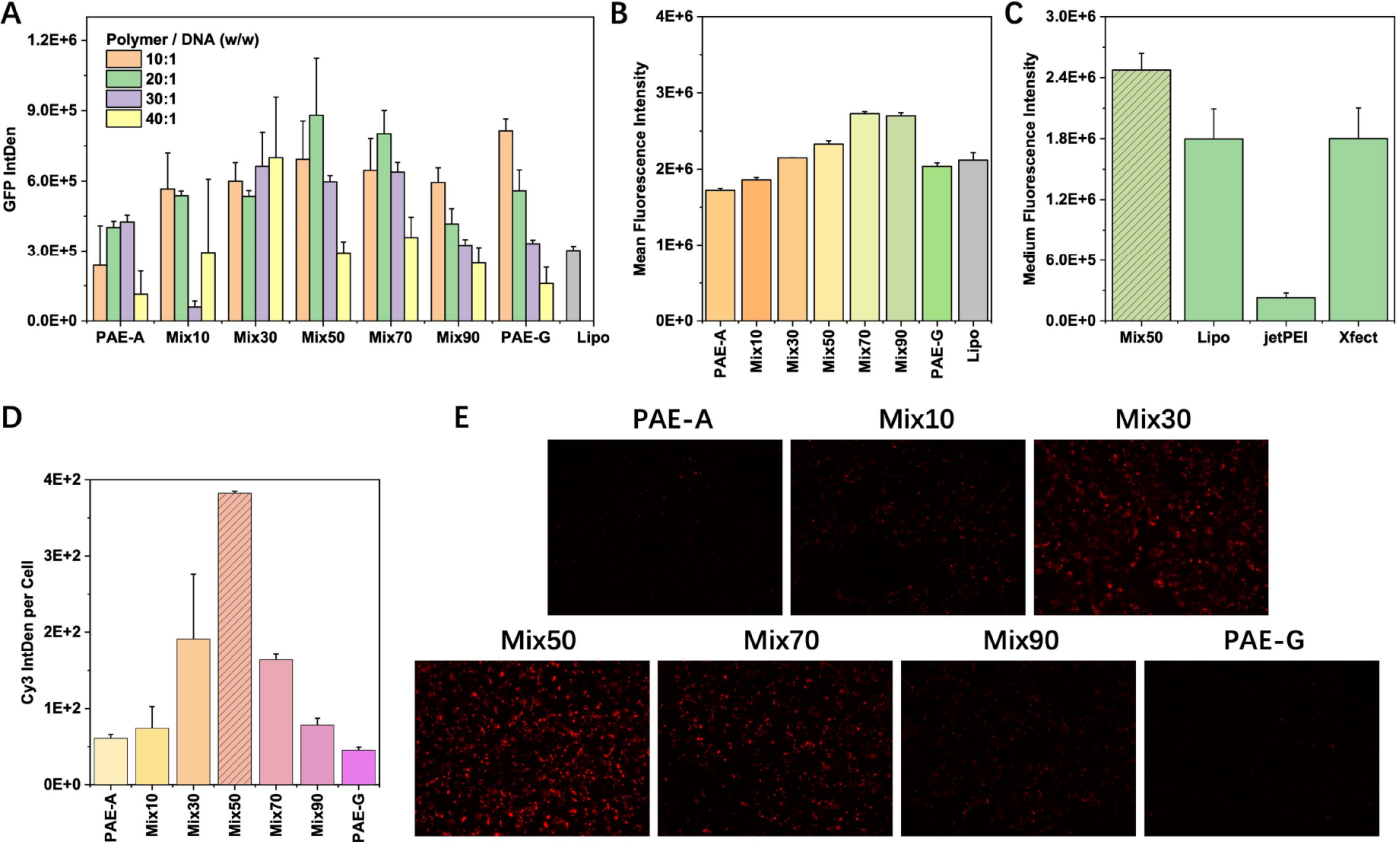
*In vitro* evaluation of DNA delivery performance of different polymers. (A) GFP expression levels in HEK cells 48 h post-transfection, with polymer-to-DNA mass ratios (w/w) set at 10:1 to 40:1. Lipofectamine 3000 (Lipo) was added as commercial control. (B) Mean Fluorescence Intensity and (C) Medium Fluorescence Intensity of GFP in HEK cells transfected with polyplexes formed with different polymers, measured by flow cytometry. Lipo, jetPEI, and Xfect were commercial control groups. (D) Average fluorescence intensity per cell 4 h after transfection with Cy3-labeled DNA using different polymers. (E) Fluorescence images of cells 4 h after transfection with Cy3-labeled DNA using different polymers. The image was taken at 40× magnification. The polymer-to-DNA mass ratio (w/w) in flow cytometry and Cy3-DNA transfection experiments was set at 20:1.

To investigate the underlying mechanism, the cellular uptake of different polyplexes by measuring the average fluorescence intensity per cell were analyzed using Cy3-labeled DNA (Figure 4 **D,** **E**). The results revealed that the cellular uptake efficiency of polyplexes followed a trend similar to the GFP expression pattern in Figure 4A, demonstrating that while both PAE-A and PAE-G can effectively deliver DNA into cells (as shown by nearly 100% transfection efficiency in all conditions), the synergistic interactions between PAE-A and PAE-G significantly enhances the efficiency of PAE-Mix50 polyplex uptake by cells.

Following this, the mRNA delivery capabilities of different polymers were investigated *in vitro*. First, TEM observations revealed that the polymer-mRNA polyplexes formed with the PAE-Mix50 vector exhibited completely different morphological features compared to those formed with PAE-A and PAE-G vectors, appearing as irregular aggregates (Figure 5A). The size of PAE-Mix50 polyplexes decreased to levels similar to that of PAE-G, while their zeta potential was closer to that of PAE-A (Figure 5B). Additionally, the stability of the mRNA-loaded polyplexes was demonstrated by monitoring changes in zeta potential and size over different incubation times (Figure S18), which showed that the polyplexes formed with PAE-Mix50 and PAE-G maintained structural stability over time, while the polyplex formed with PAE-A alone exhibited significant structural changes.

**Figure 5:**
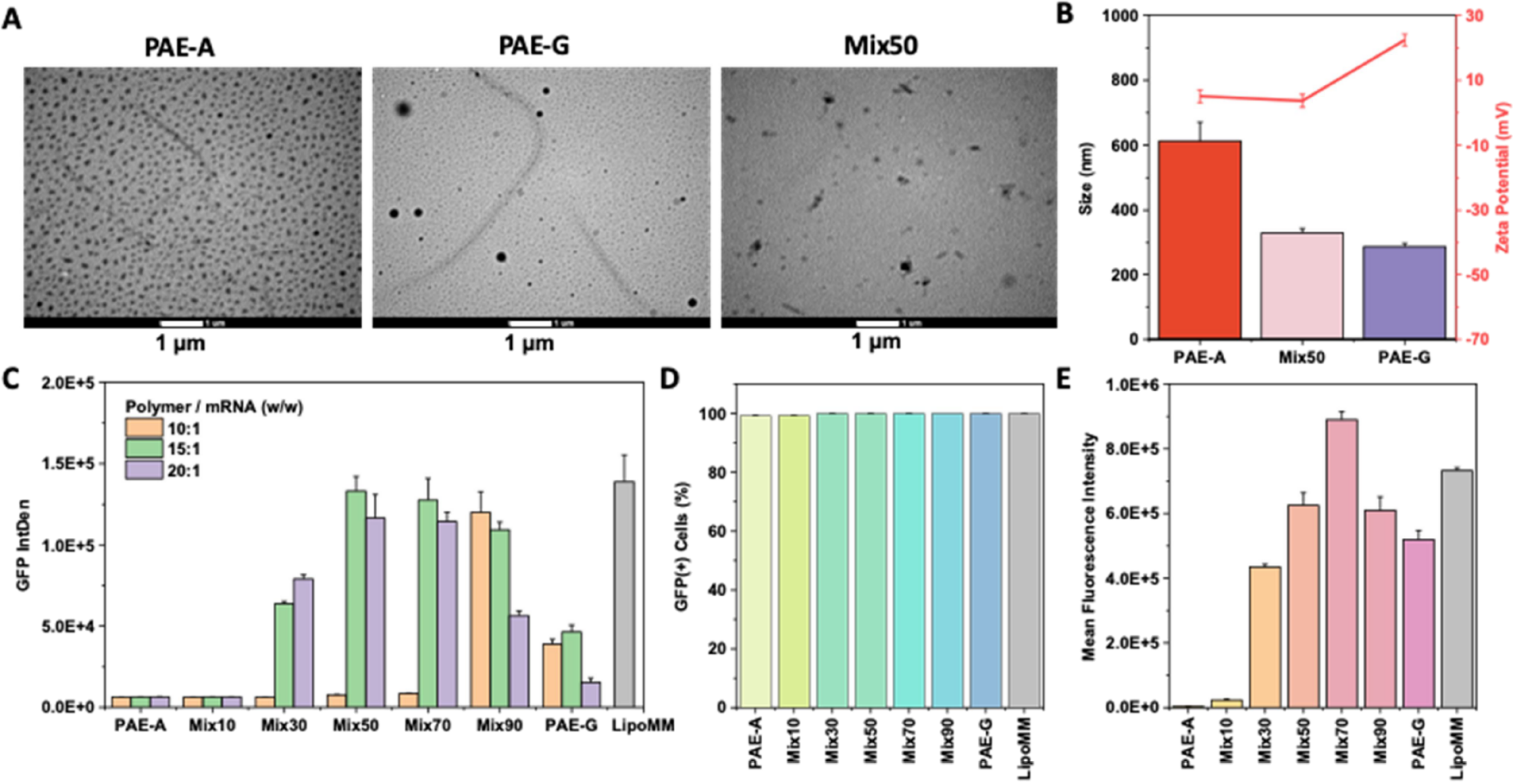
*In vitro* evaluation of mRNA delivery performance of polymers. (A) TEM images of polymer-mRNA polyplexes. The scale bar used is 1 μm. (B) Size and zeta potential of polyplexes in aqueous solution. (C) GFP expression levels in HEK cells 48 h post-transfection, with polymer/mRNA mass ratios (w/w) set at 10:1 to 20:1. (D) Percentage of GFP-positive HEK cells after mRNA transfection. (E) Mean Fluorescence Intensity of GFP in HEK cells transfected with polyplexes formed with different polymers.

In subsequent transfection experiments with HEK cells (Figure 5C), the transfection efficiency followed a trend similar to that observed for DNA delivery (Figure 4A). PAE-A alone exhibited poor mRNA delivery efficiency, however, the addition of PAE-G enhanced the delivery performance, surpassing the efficiency of PAE-G alone (∼2-fold). Remarkably, the transfection efficiency of PAE-Mix50 was comparable to that of the top commercial reagent, Lipofectamine MessengerMAX, with no notable cytotoxicity (Figure 5 **C,** Figure S19). To further quantify transfection efficiency, flow cytometry was used to evaluate both transfection rates and overall GFP expression levels. The results showed that all treated HEK cells exhibited similar degrees of GFP expression, with transfection rates approaching 100% (Figure 5 **D,** Figure S20). Notably, the fluorescence intensity of cells transfected with PAE-Mix70 polyplexes exceeded that achieved with Lipofectamine MessengerMAX (Figure 5 **E,** Figure S21).

### *In Vivo* Transfection of DNA and mRNA

In the end, PAE-A, PAE-G, and PAE-Mix polymers were tested for *in vivo* transfection via tail vein injection in mice. The mixed polymers selected for this study were the best-performing formulations from *in vitro* experiments, PAE-Mix50 and PAE- Mix70. The luciferase expression was observed at 6 and 24 h post-transfection. The 6-h time point reflected the initial expression efficiency, while the 24-h time point was used to evaluate the stability of gene expression. As can be seen in Figure 6A,B, for DNA delivery encoding luciferase, all groups exhibited peak luciferase expression at 6 h postinjection, with PAE-Mix50 showing the highest expression. Interestingly, as the PAE-A:PAE-G mixing ratio changed, not only did the fluorescence intensity vary, but the organ targeting also shifted (Figure 6C). For instance, PAE-Mix70 demonstrated pronounced spleen targeting, whereas PAE-Mix50 exhibited lung targeting. By 24 h postinjection, luciferase expression had decreased across all groups, but PAE-Mix50 still maintained a relatively high level of expression.

**Figure 6:**
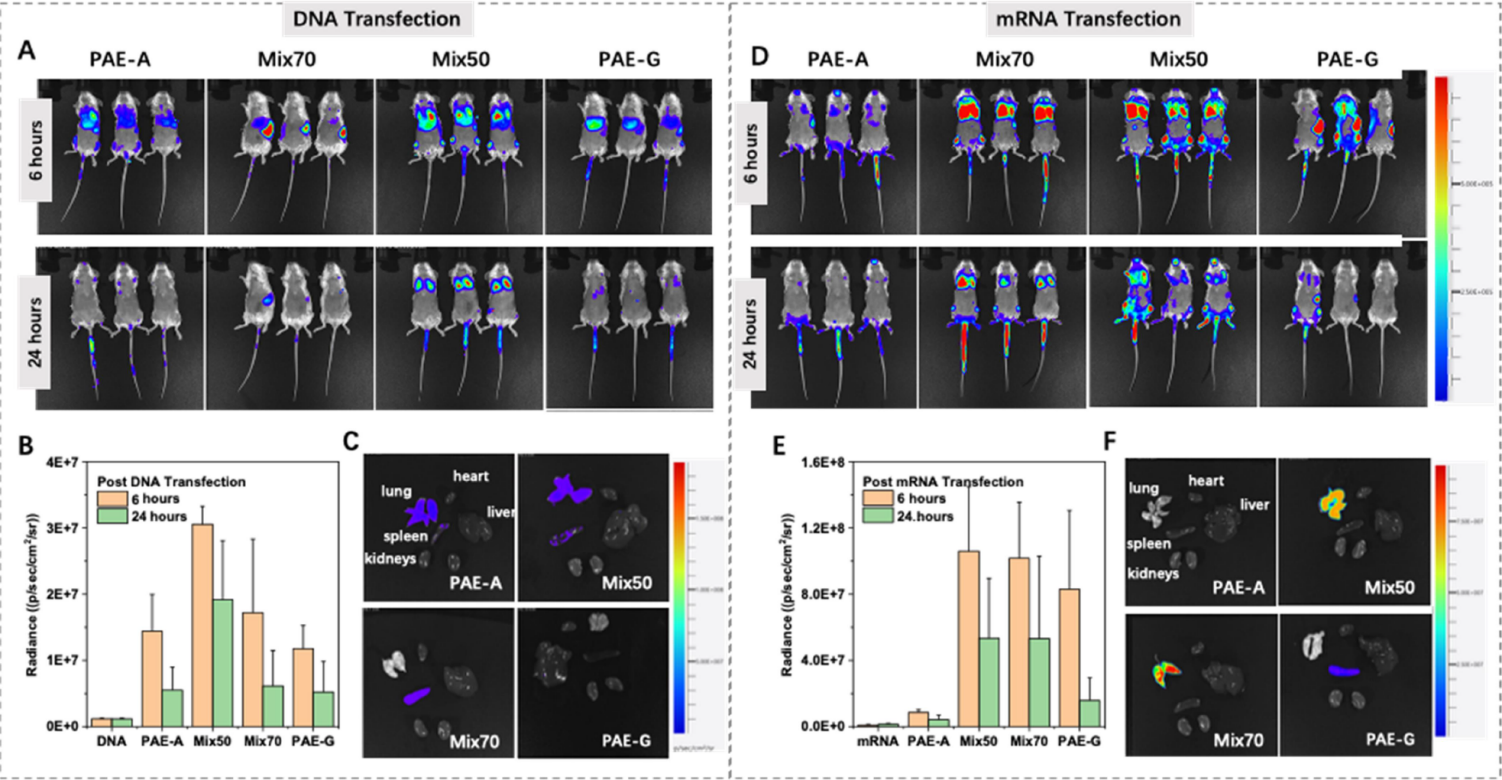
*In vivo* evaluation of delivery performance of different polymers for DNA and mRNA. (A, D) Bioluminescence images of hairless, immunocompetent mice at 6 and 24 hours after tail-vein injection of polymers complexed with (A) DNA or (D) mRNA encoding luciferase. The fluorescence intensity thresholds for A and D are the same. Naked DNA or mRNA groups are used as control. (B, E) Quantitative analysis of the corresponding bioluminescence signals from (A) and (D). (C, F) Luminescence images of major organs of mice post DNA (C) or mRNA (F) transfection.

The results for mRNA delivery were different (Figure 6D,E). Consistent with *in vitro* findings, PAE-A showed poor delivery performance. At 6 h postinjection, PAE-Mix50, PAE-Mix70, and PAE-G all exhibited strong luciferase expression. However, unlike the mixed polymers, which primarily targeted the lungs, PAE-G displayed spleen-targeting properties (Figure 6F). Overall, whether delivering DNA or mRNA, the synergistic effect of functional groups in PAE-Mix50 and PAE-Mix70 made their delivery performance outperform PAE-A and PAE-G alone.

### *In Vitro* Protein Delivery

The protein delivery capabilities of these polymers were further evaluated by delivering the toxic protein Saporin (SAP) in HEK cells (Figure S22). The results showed that all polymers were effective, with cell killing rates exceeding 70%; however, no significant differences were observed among the polymers.

## Conclusions

This study presents a novel branched PAE-based delivery platform that boosts performance by leveraging the synergistic interactions between guanidinium and tertiary amine functional groups. Three types of PAEs possessing guanidinium, tertiary amine, or both functional groups (at different ratios) were prepared. Their structures, topologies, and the interactions between guanidinium and tertiary amine groups were systematically characterized using GPC, ^1^H-, ^13^C-, and 2D-NMR, AFM, TEM, DLS, and photophysical analyses. *In vitro* and *in vivo* evaluations have demonstrated that by mixing these branched PAEs, the efficiency of polymeric vectors in nucleic acid (DNA and mRNA) delivery can be significantly enhanced (2- to 30-fold). Compared to commercial standards, the newly developed PAE vectors effectively delivers both DNA and mRNA with superior performance. This work not only deepens our understanding of structure–function relationships in polymeric vectors but also paves the way for the rational design of next-generation delivery platforms with broad biomedical implications.

## Supplementary Material



## Notes

Detailed experimental procedures, synthesis routes, NMR spectra, GPC traces measurements, absorbance and photoluminescence spectra, cytotoxicity assay, flow cytometry results, DLS measurements, stability test, pH measurement, and SAP delivery results (PDF)
