# Supplementary material for: Synergistic Effect of Guanidinium and Tertiary Amine Groups on Boosting Gene Delivery
Source: JACS Au. 2025 Jul 23;5(8):3951–9. doi: 10.1021/jacsau.5c00592 (PMC12381695; doi:10.1021/jacsau.5c00592)
Supplement: Supplementary file 1 [file au5c00592_si_001.pdf]

Supporting Information

Synergistic Effect of Guanidinium and Tertiary Amine Groups on Boosting Gene Delivery

Yinghao Li, [a],[b] Jiahao Liu, [a] Liang Yao, [b] Tianyu Mao, [c] Xinyue Wang, [a] Zhonglei He, [a]*

Jing Lyu,[b]* Wenxin Wang[a],[b]*

[a] J. Liu, X. Wang, Dr. Z. He, Prof. W. Wang
Institute of Precision Medicine (AUST-IPM)
Anhui University of Science and Technology
Huainan, China
E-mail: wenxin.wang@ucd.ie; jing.lyu@ucd.ie; hzlph@aust.edu.cn

[b] Dr. Y. Li, L. Yao, T. Dr. J. Lyu, Prof. W. Wang
Charles Institute of Dermatology, School of Medicine
University College Dublin
Dublin 4, Ireland

[c] T. Mao
Centre of Micro/Nano Manufacturing Technology (MNMT-Dublin), School of Mechanical & Materials Engineering
University College Dublin
Dublin 4, Ireland

E-mail: wenxin.wang@ucd.ie; jing.lyu@ucd.ie; hzlph@aust.edu.cn

1. Materials

For polymer synthesis and characterization, 1,4-Butanediol diacrylate (BDA), 5-amino-1-pentanol (AP), pentaerythritol tetraacrylate (PTTA), 1-Ethyl-3-(3-dimethylaminopropyl)carbodiimide (EDAC), 4-Dimethylaminopyridine (DMAP), 4-guanidinobenzoic acid hydrochloride (GAH) were purchased from Sigma-Aldrich. 1-(3-aminopropyl)-4-methylpiperazine (E7) was purchased from Fisher Scientific. Lithium bromide (LiBr) for GPC measurements was purchased from Sigma-Aldrich. Dimethyl sulfoxide (DMSO), dimethylformamide (DMF), acetone and diethyl ether were purchased from Fisher Scientific. Deuterated chloroform ($CDCl_3$) and deuterated DMSO were purchased from Sigma-Aldrich. Hank's balanced salt solution, and alamarBlue Assay Kit purchased from Sigma and Invitrogen, respectively. Lipofectamine™ 3000 (Lipo) and Lipofectamine™ MessengerMAX™ (LipoMM) were from Thermo Fisher Scientific, Xfect™ from Takara, Kusatsu, Japan. jetPEI was from Polyplus. Sodium acetate (Sigma) was diluted to 0.025 M prior to use. Picogreen was purchased from Life Technologies. Cell culture Dulbecco's modified Eagle Medium (DMEM) was purchased from Sigma. Fetal bovine serum (FBS, Gibco, was filtered through 0.2 μm filters before use. gWiz-GFP commercial plasmid were obtained from Aldevron, Fargo, ND, USA.

2. Polymer Synthesis

PAEs were synthesized via a straightforward Michael addition reaction. The progress of the reactions was monitored using Agilent 1260 Infinite gel permeation chromatography (GPC) and nuclear magnetic resonance (NMR).

PAE-A synthesis was conducted by a simple Michale Addition. Typically, BDA (3.964 g),

PTTA (0.352 g), and AP (2.269 g) were dissolved in 15 mL of DMSO and bubbled with argon for 15 minutes before the reaction was carried out at 90 °C. When the weight-average molecular weight ($M_{w,\ GPC}$) approached 10,000 Da, excess BDA (3.845 g) and 37 mL DMSO were added for initial end-capping. After 24 hours of reaction, the reaction mixture was precipitated in hexane to remove unreacted BDA. Then the purified polymer was redissolved in 53 mL of DMSO, and 3.454 g of E7 was added for final end-capping. After 24 hours of reaction, the mixture was precipitated in diethyl ether to achieve the PAE-base product. In the end, the polymer was redissolved in Acetone and precipitated in a mix solution of diethyl ether/acetone=1/1. The final product was dried under vacuum and stored at -20 ^{o}C.

For PAE-G synthesis, 1g of PAE final product, 230 mg of EDAC, 145 mg of DMAP, 258 mg of GAH were dissolved in 10 mL DMSO. After reacting at room temperature for 2 days, the polymer was purified by dialysis to remove small molecular reactants. In the end, the final polymer was precipitated in diethyl ether, dried under vacuum, and stored at -20 ^{o}C. This reaction does not follow a conventional pathway; rather, it represents a new transformation. The nitrogen of the secondary amine acts as a nucleophile to attack the O-acylisourea intermediate formed from the reaction of EDAC and the carboxylic acid. However, due to the direct linkage of the carboxyl group to the aromatic guanidine, a structural rearrangement may have occurred under its influence, leading to the departure of the E7 group from the secondary amine **(Scheme S1**).

3. Characterization Methods

Molecular Weight Measurements

Number average molecular weight ($M_{n,GPC}$), weight average molecular weight ($M_{w,GPC}$), and *Đ*

of polymers were determined by GPC equipped with a refractive index detector (RI), a viscometer detector (VS DP) and a dual angle light scattering detector (LS 15° and LS 90°). To monitor the molecular weight of polymers during the polymerization process, 20 μL of the reaction mixture was collected at different time points, diluted with 1 mL of DMF, filtered through a 0.2 μm filter and then measured by GPC. The columns (PolarGel-M, Edinburgh, UK, 7.5 mm × 300 mm, two in series) were eluted with DMF and 0.1% LiBr at a flow rate of 1 mL/min at 60 ℃. Columns were calibrated with linear poly(methyl methacrylate) (PMMA) standards.

Nuclear Magnetic Resonance (NMR)

The chemical structure and composition of polymers were confirmed with one- and two-dimensional NMR spectra of ^{1}H-NMR, ^{1}H, ^{1}H-COSY, ^{1}H, ^{1}H-TOCSY NMR. Polymer samples were dissolved in $CDCl_3$ or deuterated DMSO. Measurements were carried out on a Varian Inova 400 MHz spectrometer (Edinburgh, UK). To monitor the reaction extent during the polymerization process, 100 μL of the reaction mixture was collected at different time points, diluted with 800 μL of deuterated solvent and then measured by NMR. For Mix50 measurements, mixed polymers were first dissolved in DMSO at a concentration of 100 mg/mL, then 100 μL solution was diluted with 800 μL $CDCl_3$ for NMR measurements.

Acid Titration for Calculating Polymer pKa and Degree of Protonation

Degree of polymerization titration: 50 μL of the polymer solution is dissolved in 2 mL of water under continuous stirring. First, 10 μL of 1 M NaOH solution is added to ensure deprotonation of the polymer. The titration is then carried out using 0.1 M HCl solution while continuously monitoring the pH of the solution. The pKa value is determined as the pH at the midpoint of

the titration curve, after correcting for the initial addition of NaOH. The degree of protonation of the polymer can be calculated using the Henderson-Hasselbalch equation: pH=pKa+log([A-]/[HA]).

By rearranging the equation, the ratio of protonated to deprotonated species is obtained, and the degree of protonation is expressed as [HA]/[A-]*100%. This method provides an accurate way to evaluate the protonation behavior of the polymer in solution.

Polyplex Preparation

Generally, the polymers were initially dissolved in DMSO to stock solutions (100 mg/mL), and then the stock solutions were further diluted with 25 mM sodium acetate buffer according to the w/w ratio. As an example, for polymer/ DNA =20:1, the polymers were diluted to 2 mg/mL. DNA was diluted to 0.1 mg/mL with sodium acetate buffer. The polymer solutions were added into the DNA solution at equal volume, vortexed for 10 s, and allowed to stand for 15 min.

Size and Zeta Potential Assessment

The polyplex was prepared as mentioned above. After that, the sizes and zeta potentials of polyplexes were measured with a Malvern Panalytical Zetasizer (ZTS1240). All the measurements were repeated three times.

Picogreen Assays

The polyplex were prepared as mentioned above. 2 μg of DNA was used for each sample preparation. Then, 60 μL of Picogreen solution, which was prepared according to supplier's instructions, was added and allowed to incubate for another 5 min. To a 96-well plate, 200 μL of medium (without serum) or water was added, and then 30 μL of the polyplex solution was added. Fluorescence measurements were carried out with a plate reader with an excitation at

490 nm and emission at 535 nm. DNA binding efficiency (BE) was calculated as: $BE = {(F_{DNA} - F_{Sample})}/{(F_{DNA} - F_{Blank})}$.

F_{DNA} was the florescence measurement of free DNA without polymer, F_{Sample} was the florescence of a polyplex at a given weight ratio between polymer to DNA, and F_{Blank} was the florescence from PicoGreen working solution only with the buffer used for polyplex formulation.

PVD protocol

The samples were mounted on the holder and placed into the chamber. The pump was then turned on to lower the pressure to 6×10^{-6} mbar, followed by introducing argon gas to bring the pressure to 5×10^{-3} mbar. Next, the Ti shutter and QCM shutter were opened, and the DC power supply was activated, depositing a 5 nm Ti layer onto the samples' surfaces. Finally, the DC power supply, argon gas, and pump were turned off. Once the pressure reached 10^3 mbar, the samples were removed from the chamber.

Atomic Force Microscopy (AFM): Polymer Morphology Study

The morphologies of the samples were analyzed using atomic force microscopy (AFM) under peak force tapping mode. All AFM images were captured with a Bruker AFM system equipped with single carbon diamond cantilever tips (Adama Innovations), featuring a nominal spring constant of 2.8 N m^{-1} and a resonance frequency of 65 kHz. The images were acquired at a scan rate of 1 Hz over a scan area of 5 µm × 5 µm. Non-functionalized tips were used for all measurements to ensure accurate assessment of surface topography without introducing additional interactions.

Transmission Electron Microscopy (TEM)

The polyplex was prepared as described above. Then, 1 μL of the polyplex solution was added to 1 mL of pure water and vortexed to ensure uniform dispersion. A 10 μL aliquot of the diluted solution was dropped onto a copper grid and air-dried. TEM imaging was performed using a TECNAI 12 instrument with an exposure time of 2.6 seconds.

Cell Culture

Human Embryonic Kidney 293 cells (HEK293) and human cervical cancer cells (HeLa) were cultured in Dulbecco's modified Eagle Medium high glucose containing 10% fetal bovine serum and 1% Penicillin-Streptomycin. Cells were cultured at 37 ℃ with 5% CO_2 in a humid incubator, under standard cell culture techniques.

Cytotoxicity Assessment (alamarBlue assay)

To perform alamarBlue assay, cell supernatants were first removed and then cells were washed with HBSS, followed by the addition of 10% alamarBlue reagent in HBSS. Living, proliferating cells maintain a reducing environment within the cytosol of the cell, converting the non-fluorescent ingredient resazurin in alamarBlue to the highly fluorescent compound resorufin. This reduction results in a color change from blue to light red and allows for the quantitative measurement of cell viability based on the increase in overall fluorescence and color of the media. The alamarBlue solution from each well was transferred to a fresh flat-bottomed 96-well plate for fluorescence measurements at 590 nm. Control cells without any treatment were used to normalize the fluorescence values and plotted as 100% viable.

Polyplex Cellular Uptake

GFP DNA was labelled with a Cy3 (a red fluorescent dye) labelling kit as per the recommended protocol. In 96-well plates, HEK cells were seeded. Gene transfection was conducted as above

with 2.5 μg/mL of DNA. After 4 hours, the medium was removed, and cells were washed with PBS three times and incubated with Hoechst 33342 for 30 mins. Fluorescent images of Cy3 and DAPI were visualized under a fluorescence microscope (Olympus IX81). The intensity of Cy3 fluorescence and cells number in each image were analyzed using the ImageJ Fiji software (NIH, Bethesda, MD, USA).

Cell Transfection

Cells were transfected with polyplexes prepared as described above, mixed with the cell culture medium, and added to cells at DNA/mRNA concentration of 5 μg/mL. Commercial reagents were used for transfection according to their protocol. Expression of the GFP reporter gene green fluorescent protein (GFP) was visualized 48 hours after transfection using an Olympus IX81 fluorescence microscope. The intensity of GFP fluorescence was analysed and semi-quantified using the ImageJ Fiji software.

Polyplex Cellular Uptake

GFP DNA was labelled with a Cy3 (a red fluorescent dye) labelling kit as per the recommended protocol. In 96-well plates, HEK cells were seeded. Gene transfection was conducted as above with 2.5 μg/mL of DNA. After 4 hours, the medium was removed, and cells were washed with PBS three times and incubated with Hoechst 33342 for 30 mins. Fluorescent images of Cy3 and DAPI were visualized under a fluorescence microscope (Olympus IX81). The intensity of Cy3 fluorescence and cells number in each image were analyzed using the ImageJ Fiji software.

Animal studies

All animal procedures were conducted in accordance with protocols approved by the

Biomedical Research Ethics Committee of Anhui University of Science and Technology (Approval No. SZ2024-004). Female BALB/c mice (8-10 weeks old, 18-22 g body weight) were obtained from Hangzhou Ziyuan Laboratory Animal Technology Co., Ltd. and housed under standard specific pathogen-free (SPF) conditions with ad libitum access to food and water. The nanoparticles, with a Polymer to DNA ratio of 20μg/1μg and a DNA concentration of 300μg/mL, were intravenously administered via tail vein injection at a volume of 200μL per mouse. Bioluminescence imaging was performed using the Tanon ABL-X5 in vivo imaging system, with mice receiving an intraperitoneal injection of 150 μL D-luciferin (30 mg/mL in PBS) at 6 and 24 hours post-nanoparticle injection. Signal intensities were quantified using Tanon Image Analysis Software (version Tanon Prime-5000 In Vivo Imaging System).

4. Experiment Data

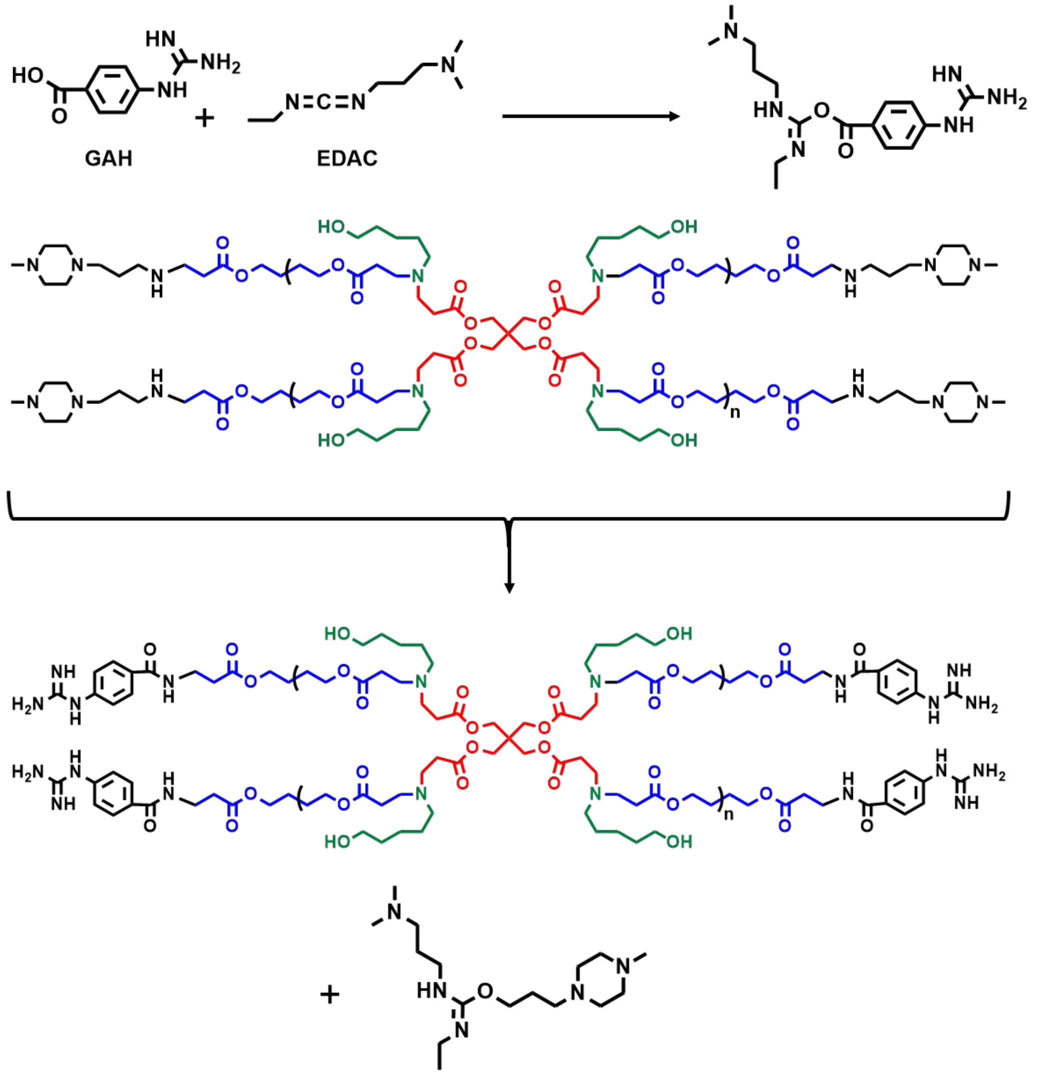

Scheme S1. Schematic diagram of the synthetic routes for PAE-G.

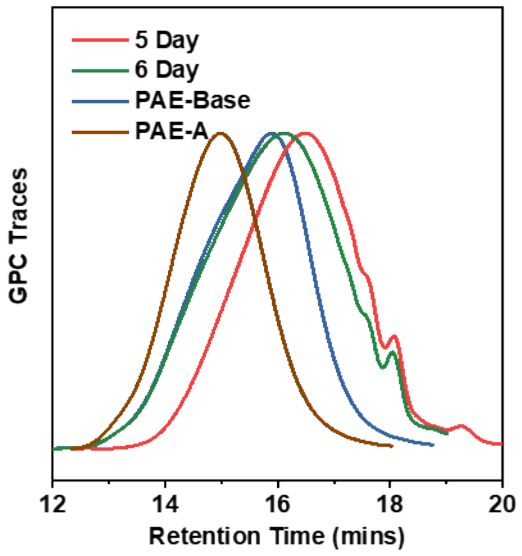

Figure S1. GPC traces of PAE-A synthesis. PAE-Base is the crude product after initial precipitation in diethyl ether.

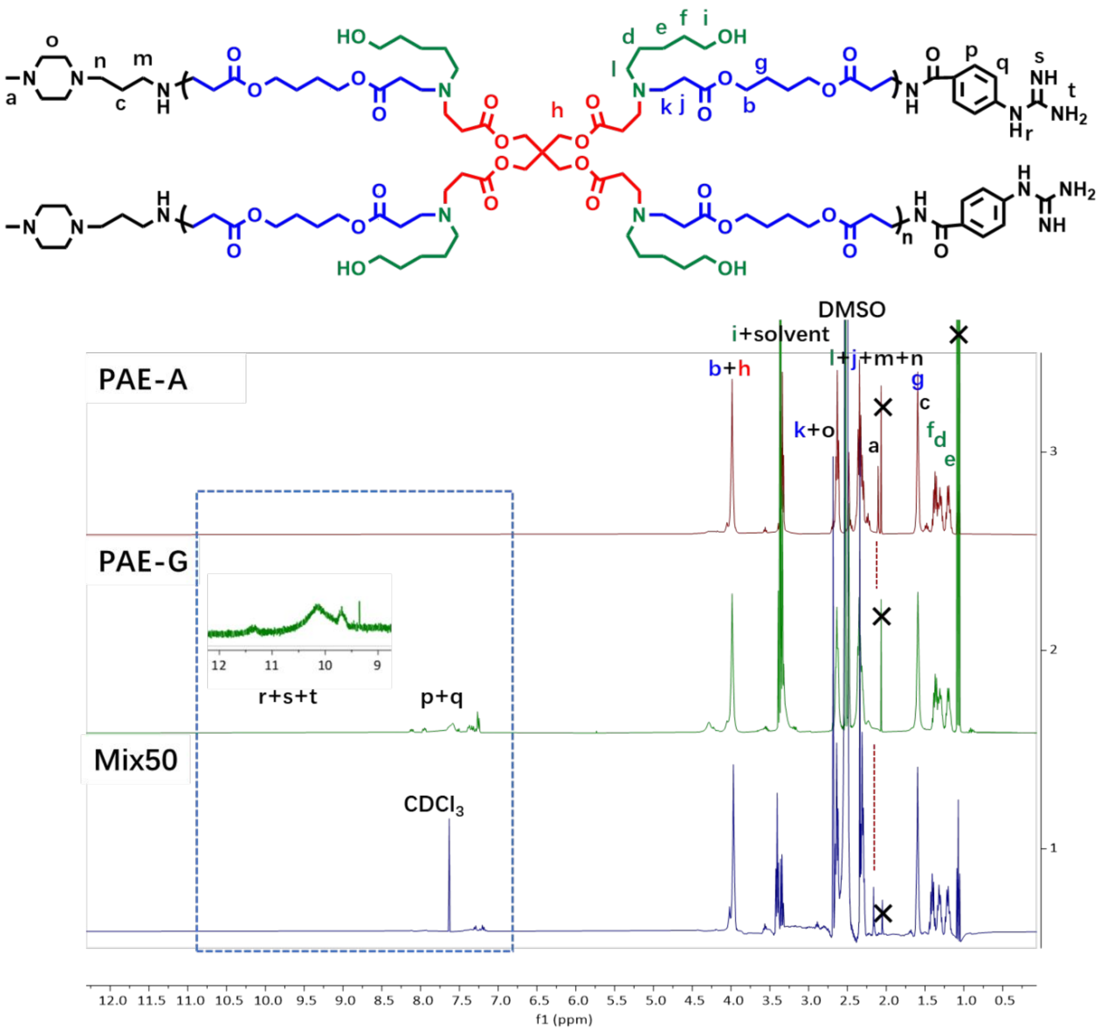

Figure S2. ^{1}H NMR spectra of PAE-A, PAE-G, and PAE-Mix50. The structure shown is only for indicating the characteristic functional groups on different polymers. PAE-A consists solely of the left-side structure, while PAE-G contains only the right-side structure. PAE-Mix50 is a physical blend of PAE-A and PAE-G, rather than a single polymer incorporating both functional groups chemically. "X" indicates that the peak corresponds to a residual solvent (acetone or diethyl ether). PAE-A and PAE-G were dissolved in deuterated DMSO. Mix50 was dissolved in $CDCl_3$.

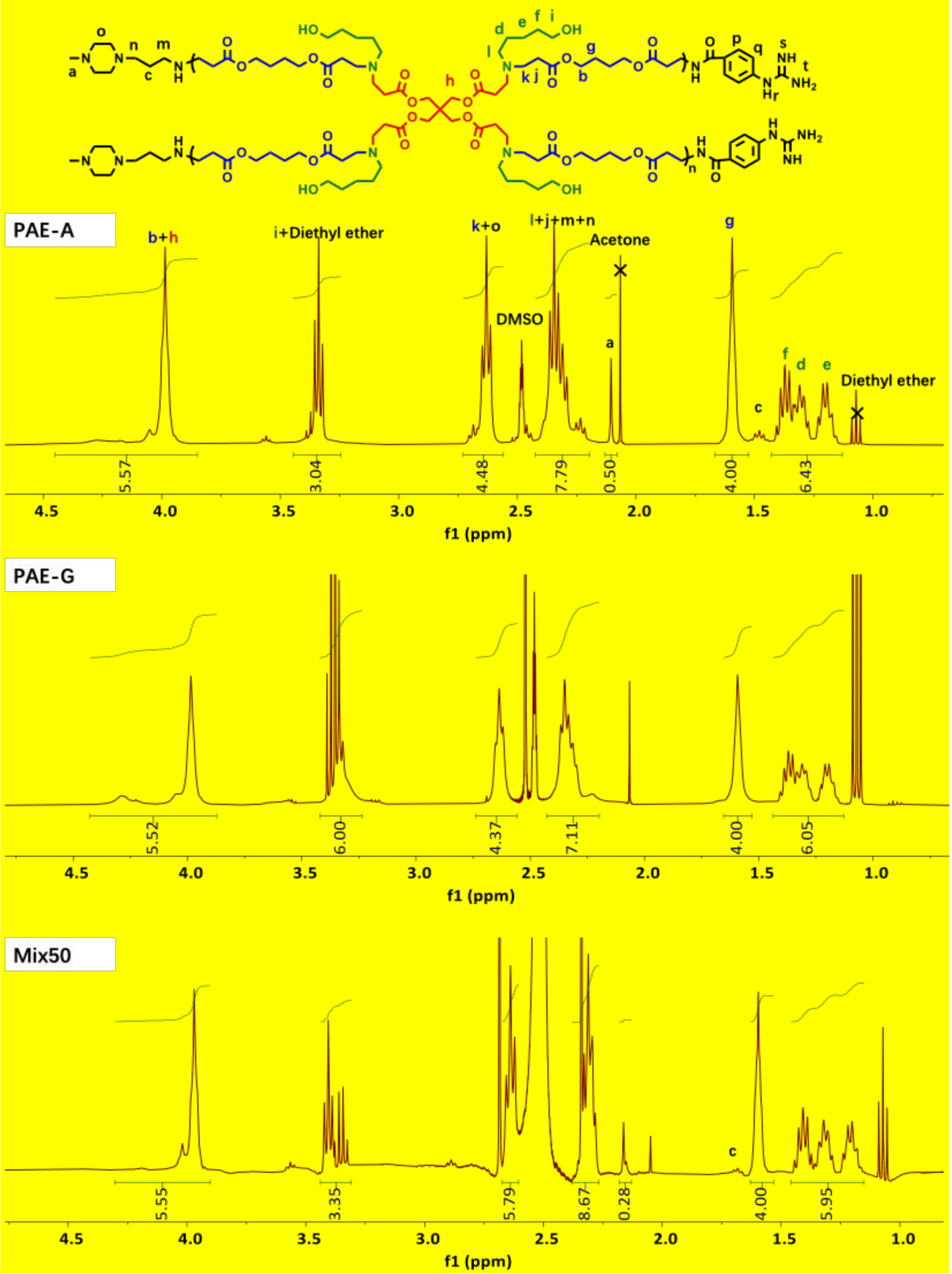

Figure S3. ^{1}H NMR spectra of PAE-A, PAE-G, and PAE-Mix50 from 1.0 to 4.5 ppm.

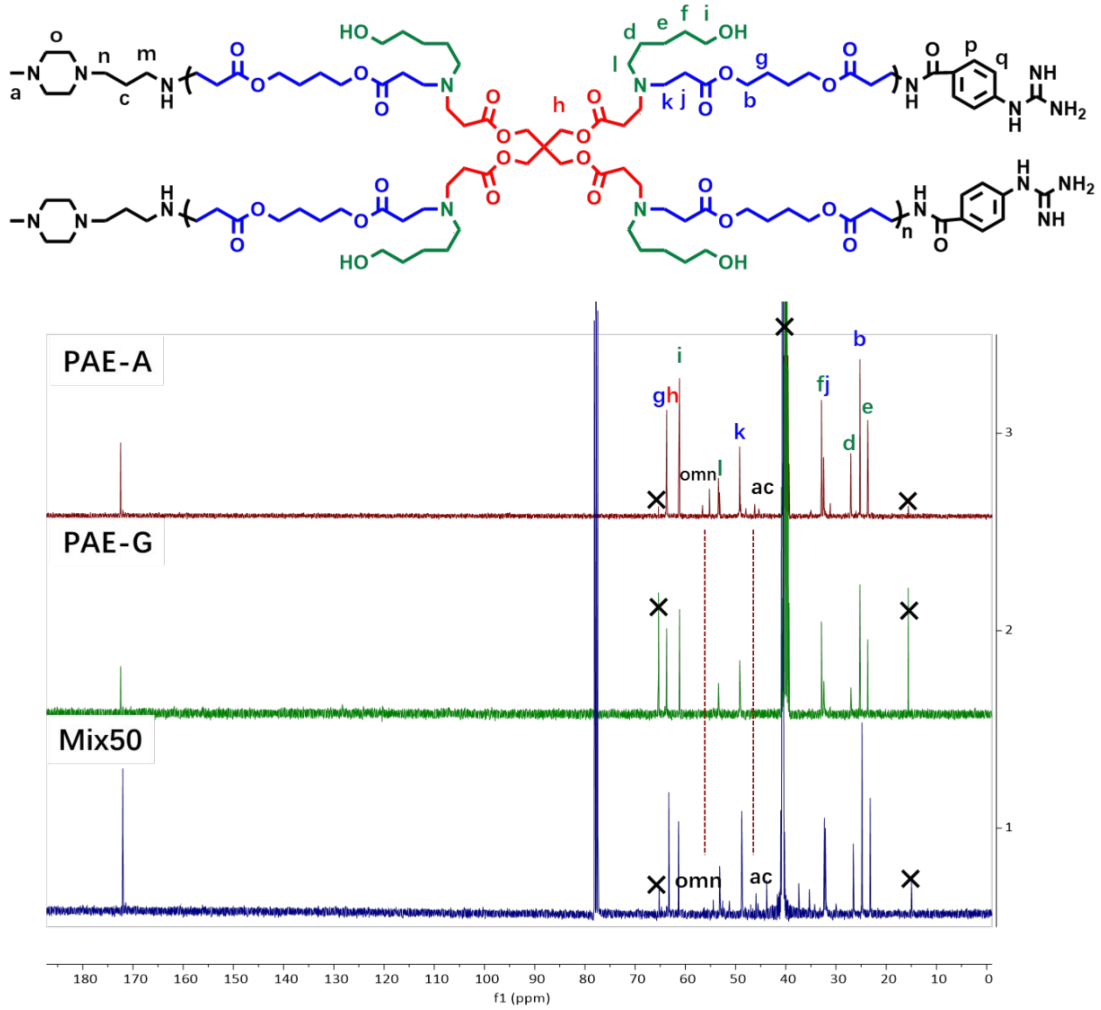

Figure S4. ^{13}C NMR spectra of PAE-A, PAE-G, and PAE-Mix50. The structure shown is only for indicating the characteristic functional groups on different polymers. PAE-A consists solely of the left-side structure, while PAE-G contains only the right-side structure. PAE-Mix50 is a physical blend of PAE-A and PAE-G, rather than a single polymer incorporating both functional groups chemically. "X" indicates that the peak corresponds to a residual solvent (acetone or diethyl ether). PAE-A and PAE-G were dissolved in deuterated DMSO. Mix50 was dissolved in $CDCl_3$.

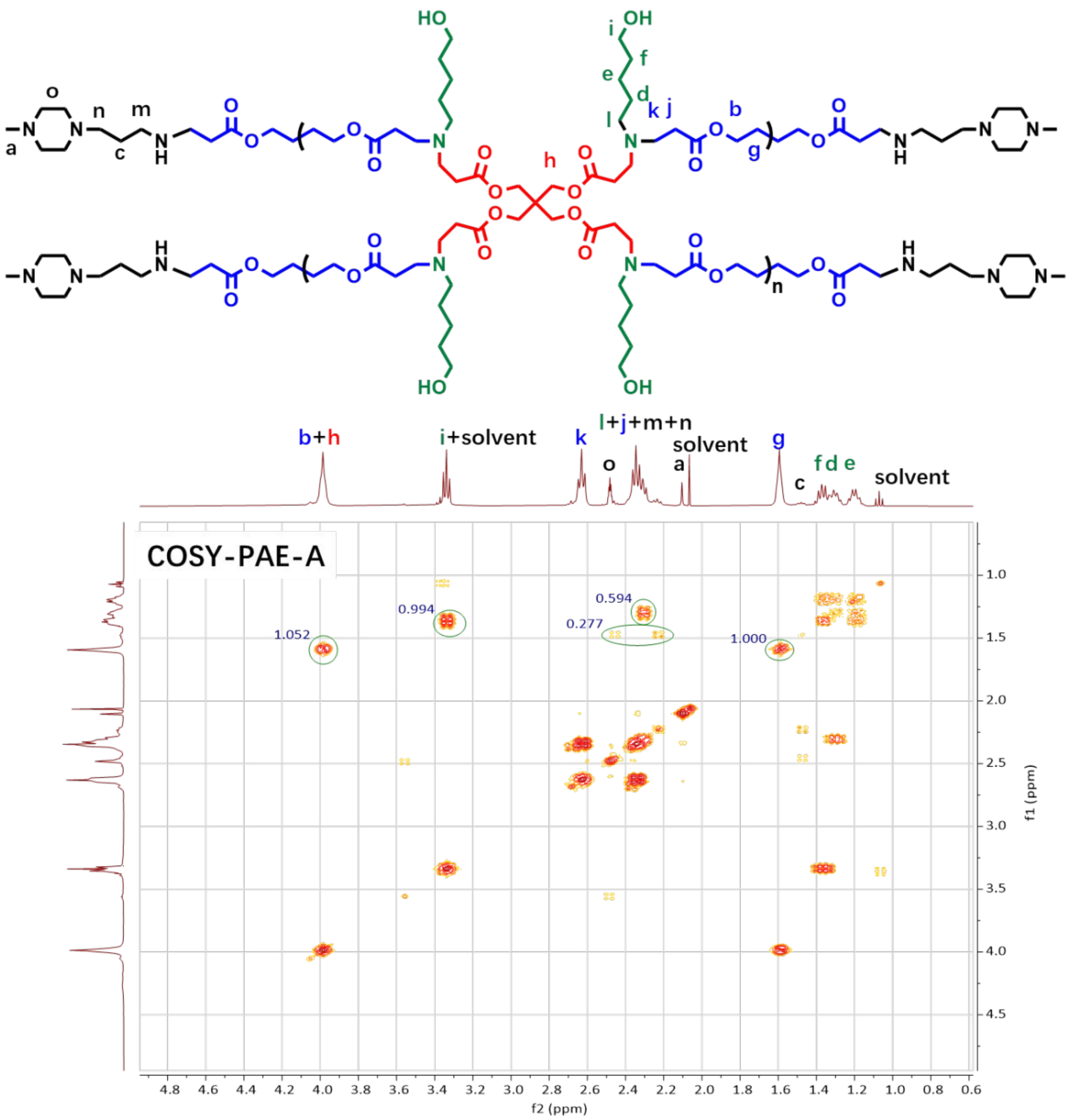

Figure S5. ^{1}H, ^{1}H -COSY spectrum of PAE-A. PAE-A was dissolved in deuterated DMSO.

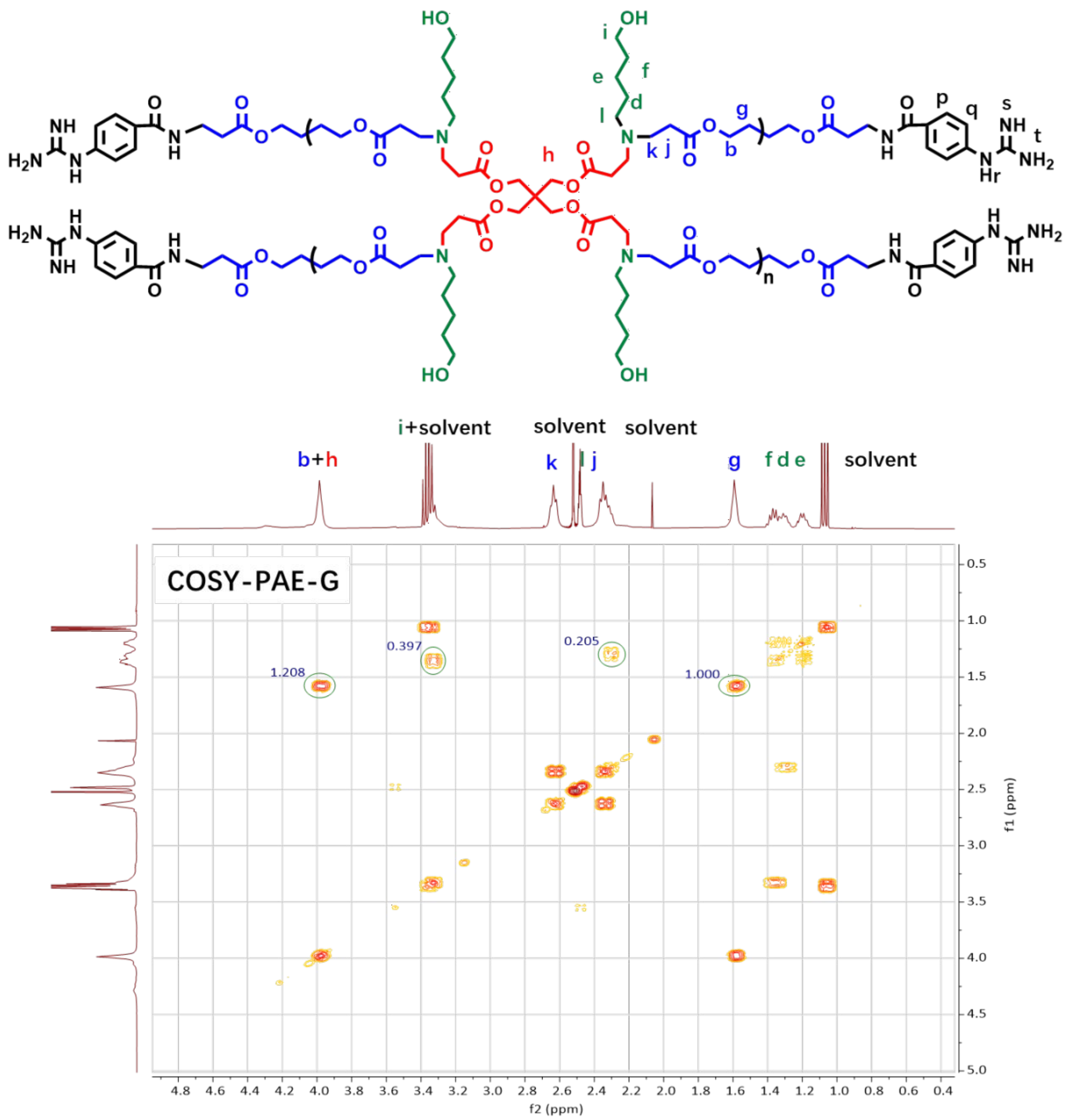

Figure S6. ^{1}H, ^{1}H -COSY spectrum of PAE-G. PAE-G was dissolved in deuterated DMSO.

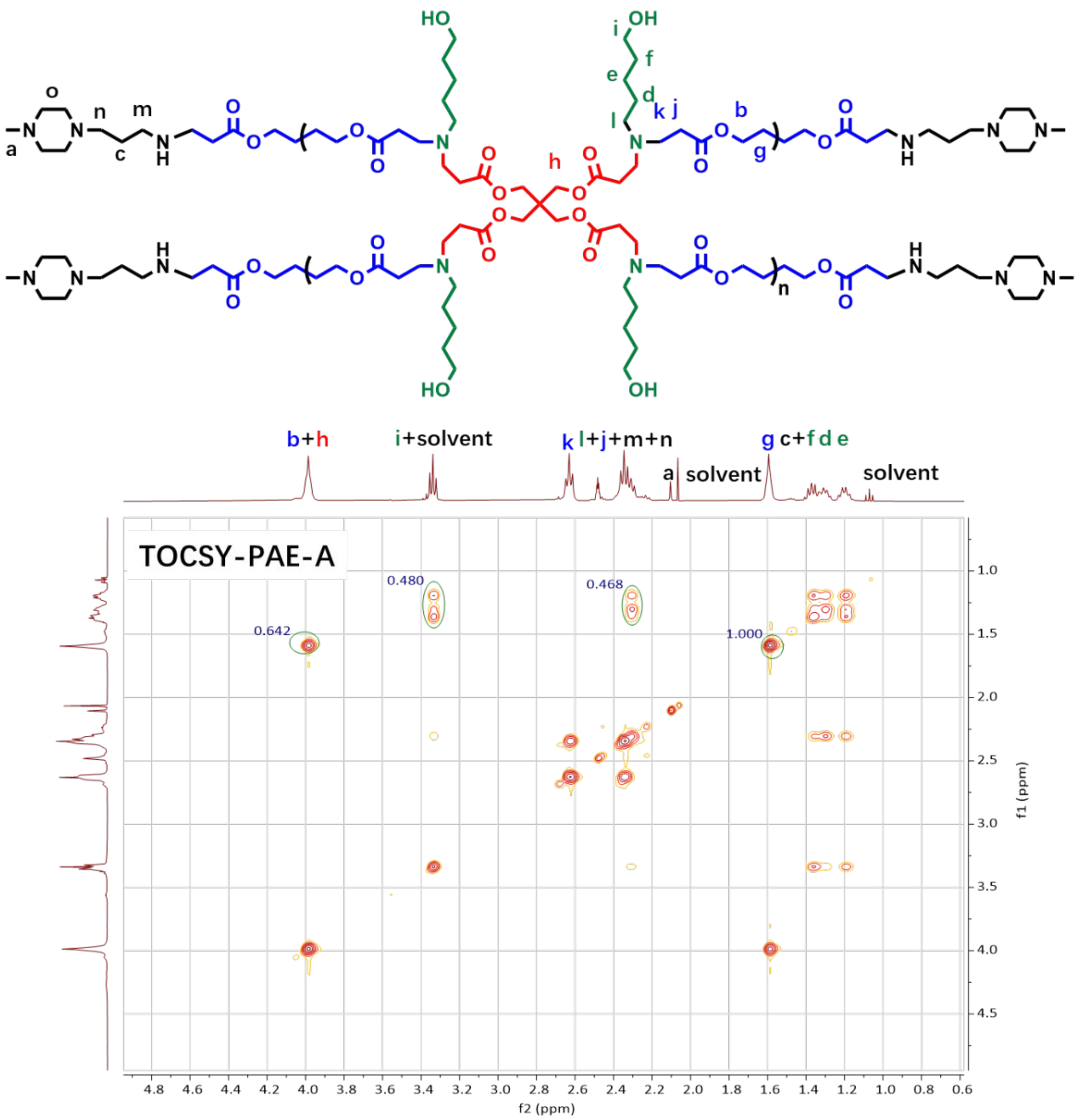

Figure S7. ^{1}H, ^{1}H -TOCSY spectrum of PAE-A. PAE-A was dissolved in deuterated DMSO.

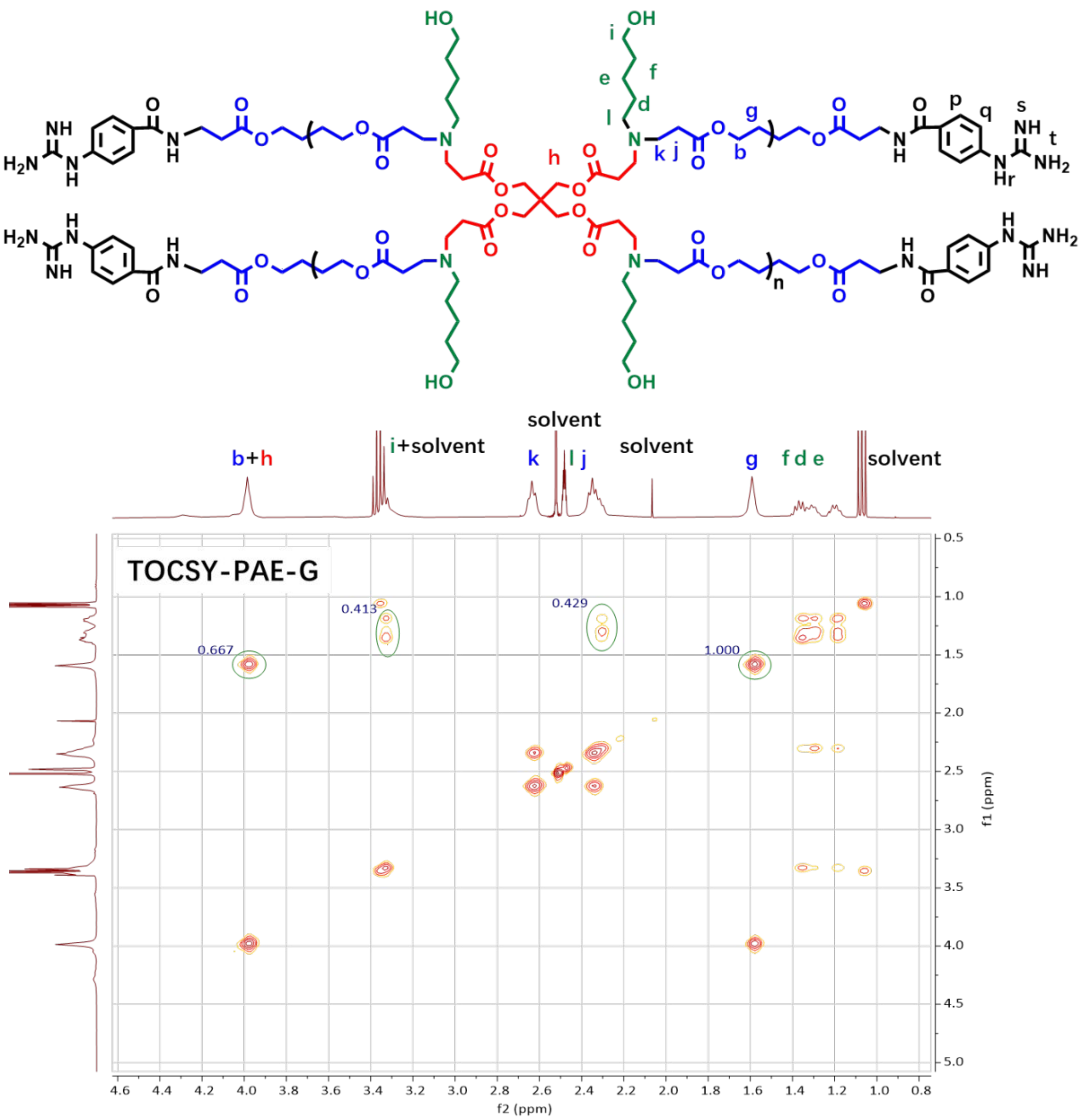

Figure S8. ^{1}H, ^{1}H -TOCSY spectrum of the PAE-G. PAE-G was dissolved in deuterated DMSO.

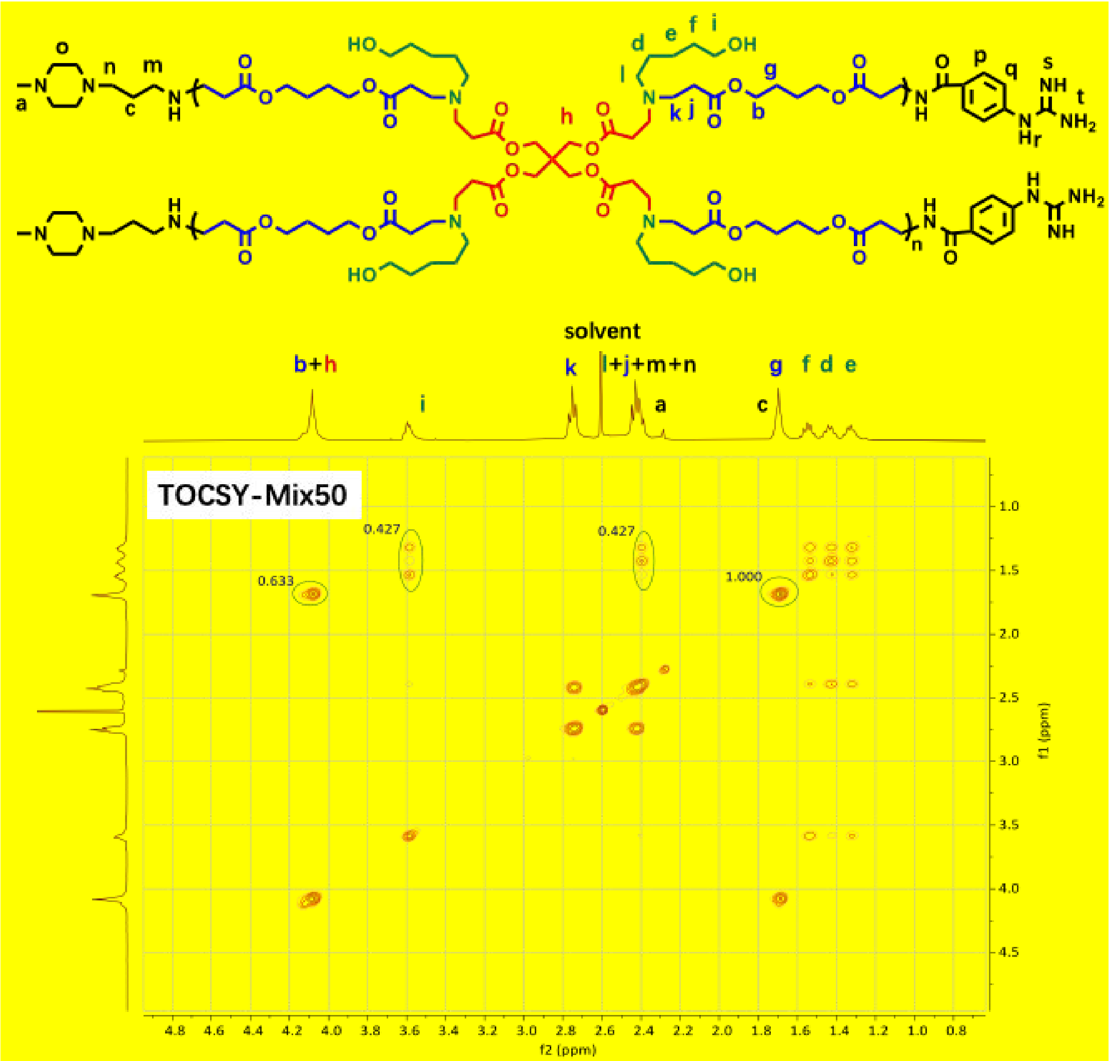

Figure S9. ^{1}H, ^{1}H -TOCSY spectrum of the PAE-Mix50. The structure shown is only for indicating the characteristic functional groups on different polymers. PAE-Mix50 is a physical blend of PAE-A and PAE-G, rather than a single polymer incorporating both functional groups chemically. Mix50 was dissolved in $CDCl_3$.

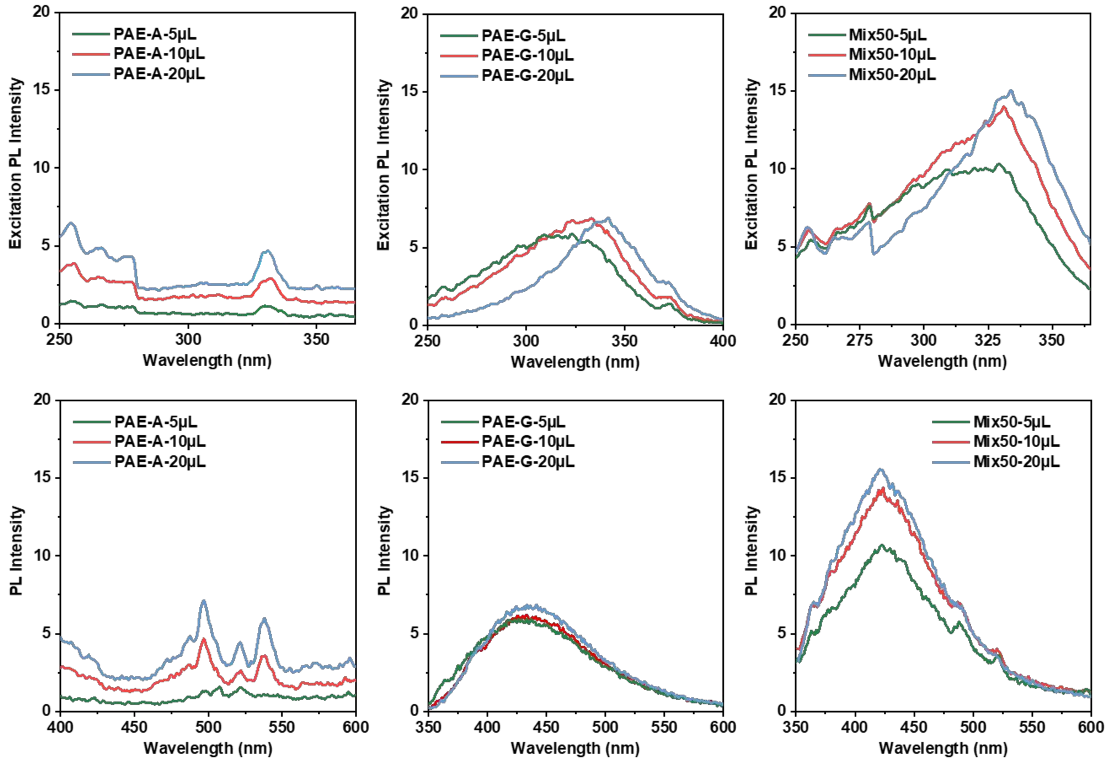

Figure S10. Absorption and emission spectra at different polymer concentrations. Measurements were conducted by dissolving 5–20 μL of a 100 mg/mL polymer stock solution in 3 mL pure water.

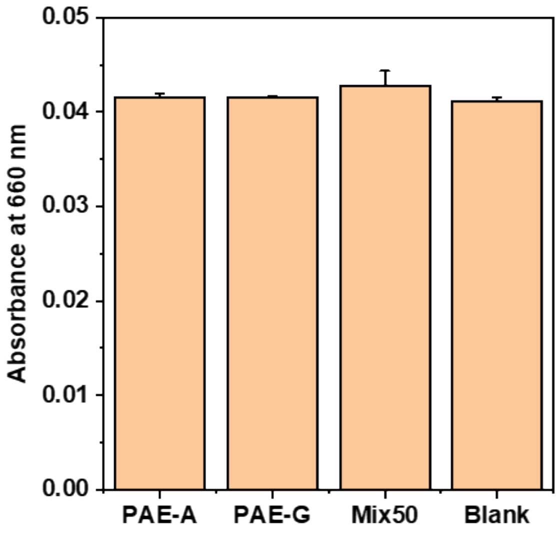

Figure S11. Absorbance of polymers in water at 660 nm. Measurements were conducted by dissolving 10 μL of a 100 mg/mL polymer stock solution in 100 μL of pure water. The blank control group consisted of pure water.

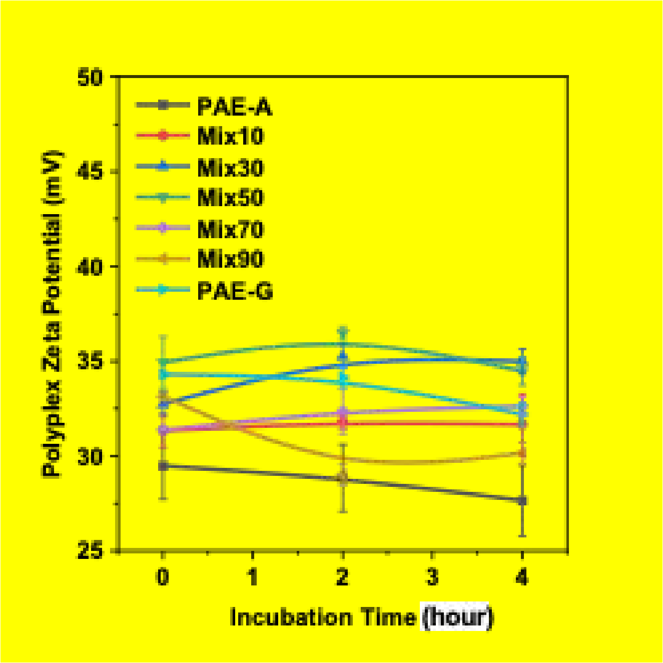

Figure S12. Changes in the zeta potential of polymer-DNA polyplexes during incubation at 37°C over time.

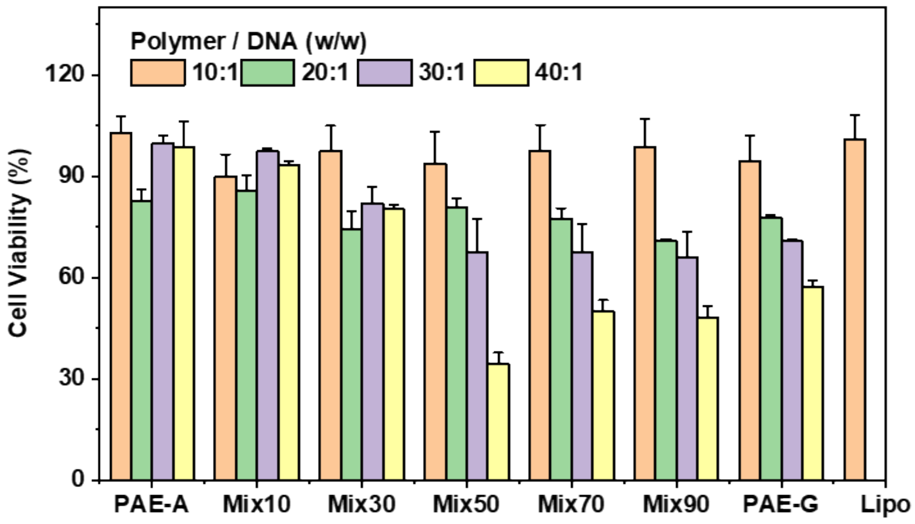

Figure S13. Cell viability post 48 hours transfection by polymer-DNA polyplexes. The commercial control group Lipo is Lipofectamine 3000.

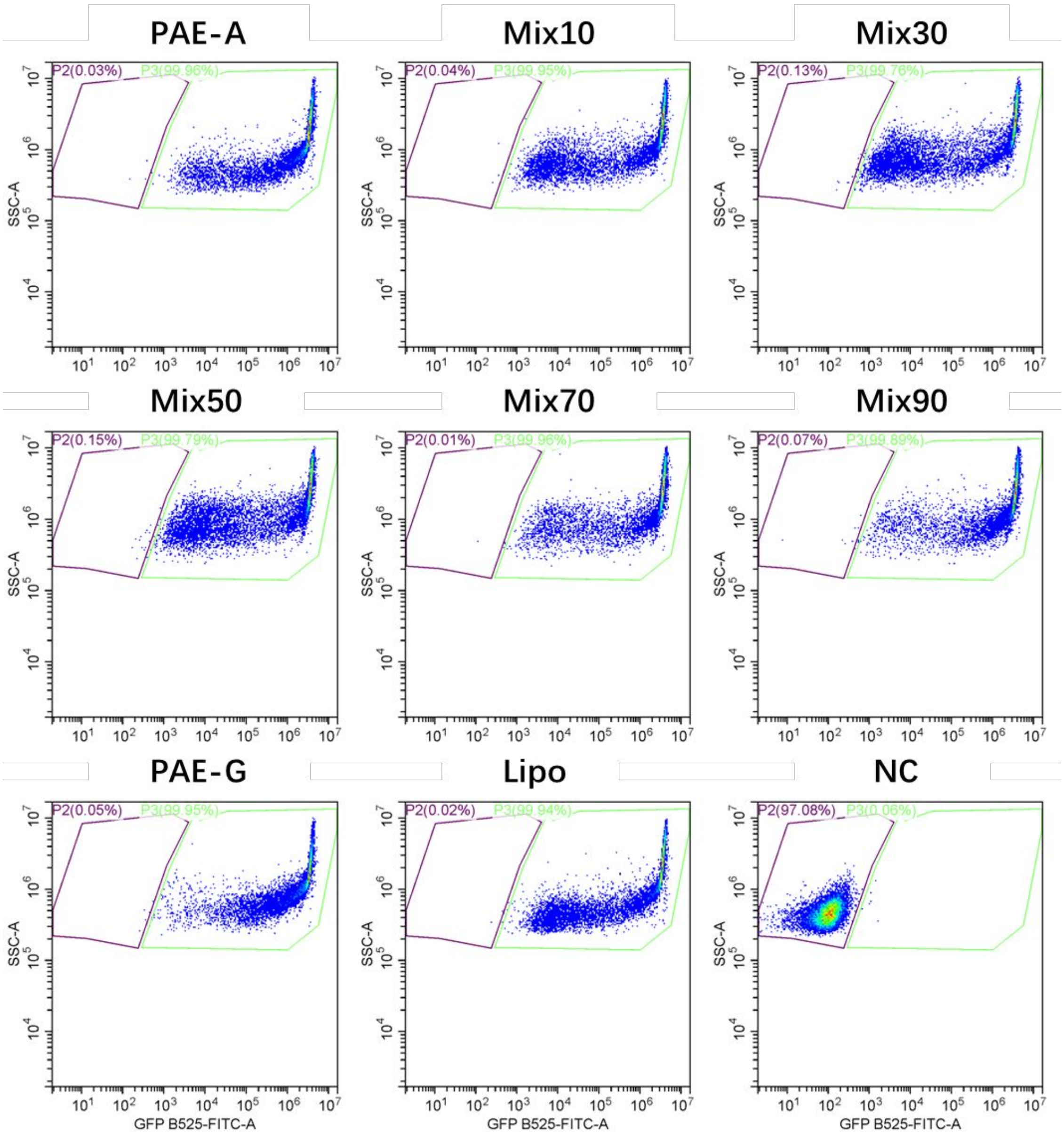

Figure S14. GFP-positive cells gating strategy for flow cytometry post 48 hours transfection by polymer-DNA polyplexes. The commercial control group Lipo is Lipofectamine 3000. NC is untreated group.

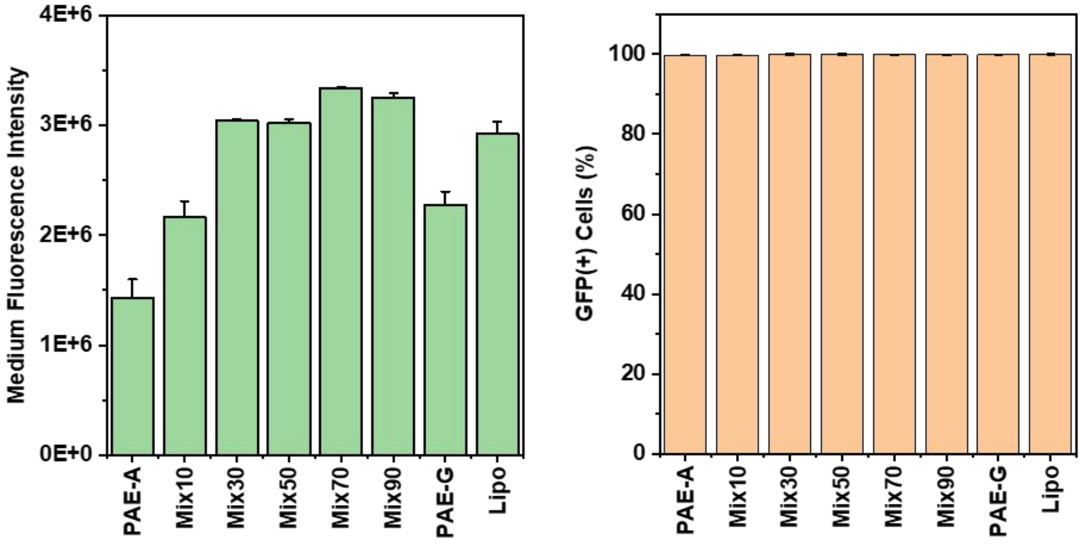

Figure S15. Percentage of GFP-positive HEKs and the medium fluorescence intensity of cells after DNA transfection.

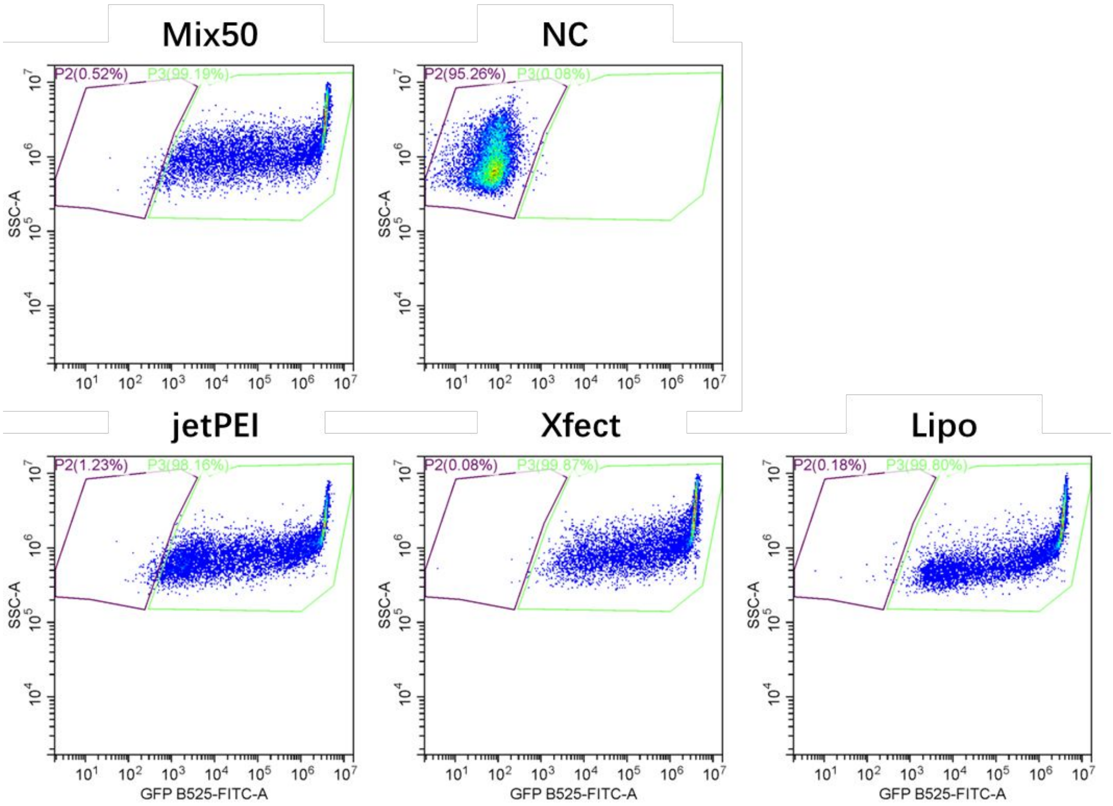

Figure S16. GFP-positive cells gating strategy for flow cytometry post 48 hours transfection by different trans agents. The commercial control group Lipo is Lipofectamine 3000. NC is untreated group.

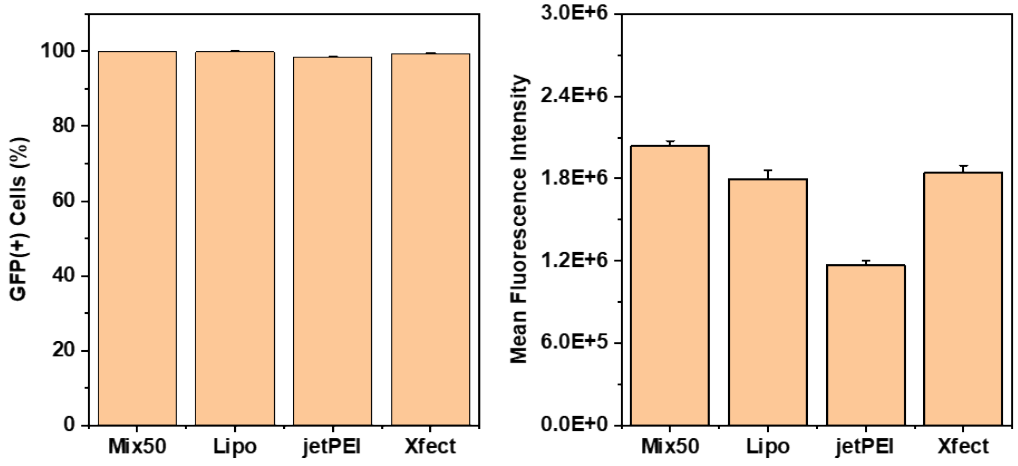

Figure S17. Percentage of GFP-positive HEKs and the mean fluorescence intensity of cells after DNA transfection by different trans agents. Lipo (Lipofectamine 3000), jetPEI, and Xfect were commercial trans agents.

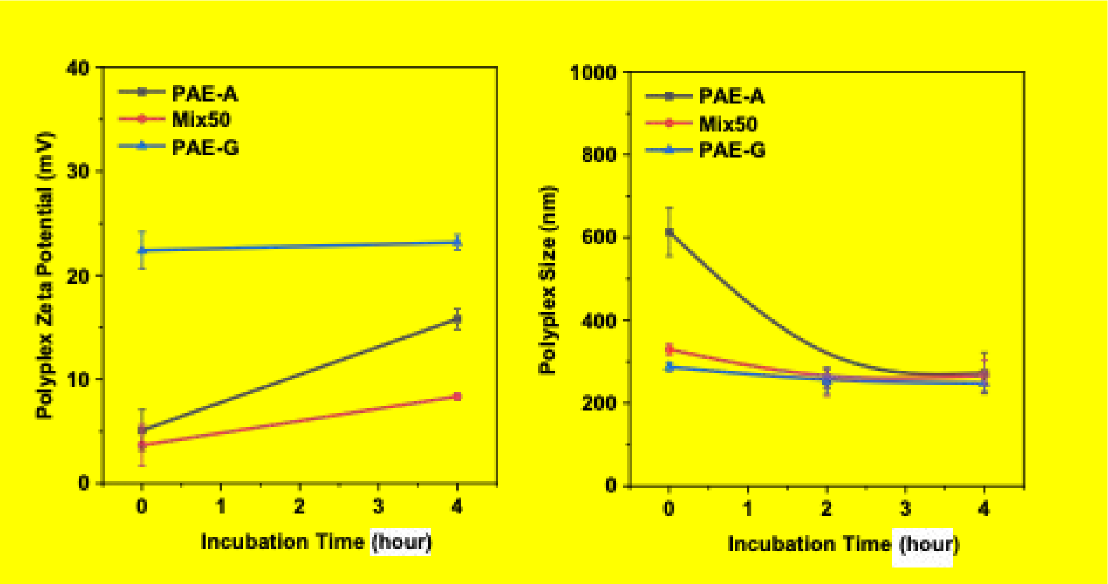

Figure S18. Particle size and zeta potentials of different polymer/mRNA polyplexes over different incubation time at 37°C at polymer/mRNA weight ratio (w/w) of 20:1.

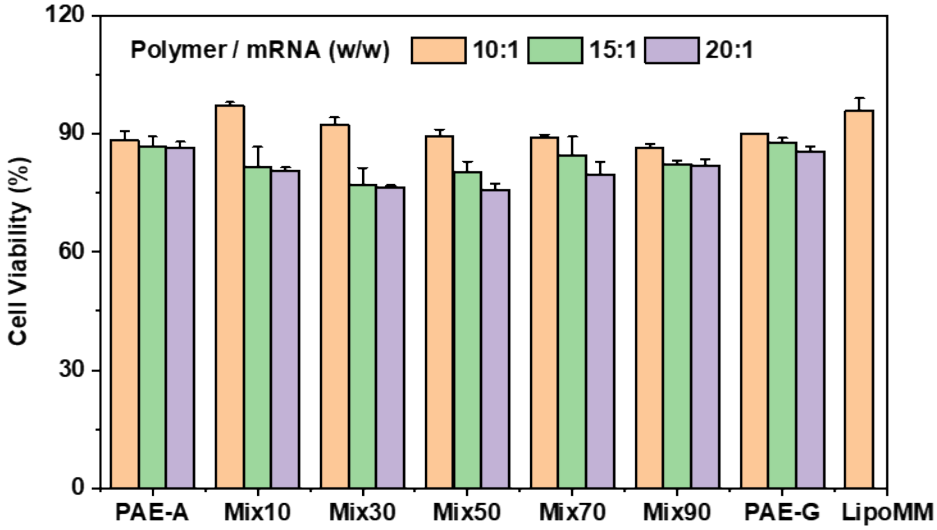

Figure S19. Cell viability post 48 hours transfection by polymer/mRNA polyplexes. The commercial control group LipoMM is Lipofectamine MessengerMAX.

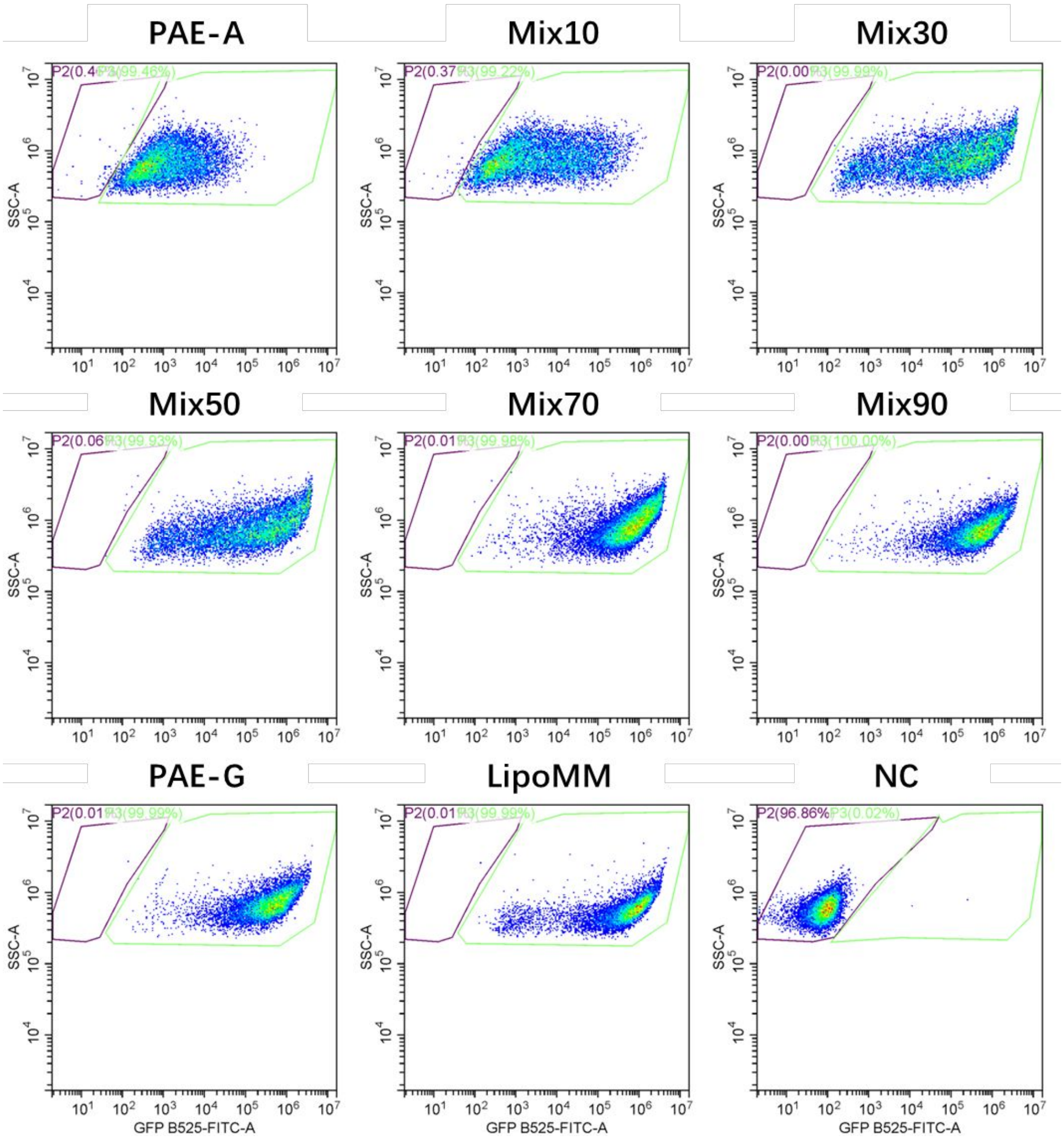

Figure S20. GFP-positive cells gating strategy for flow cytometry post 48 hours transfection by different trans agents. The commercial control group LipoMM is Lipofectamine MessengerMAX. NC is the untreated cells.

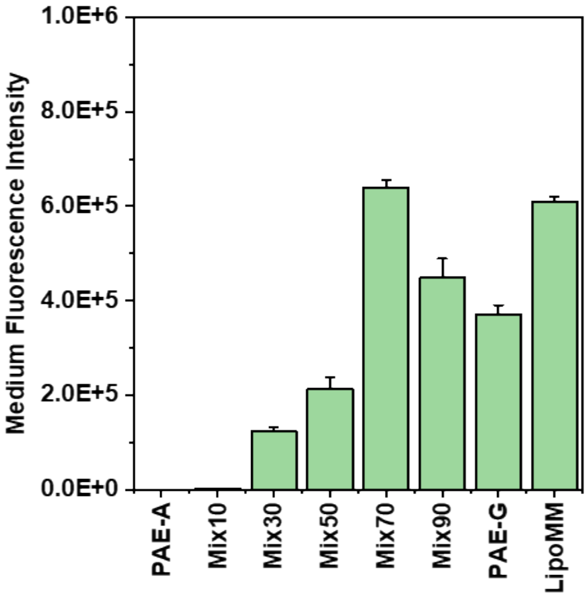

Figure S21. Percentage of GFP-positive HEKs and the medium fluorescence intensity of cells after mRNA transfection. The commercial control group LipoMM is Lipofectamine MessengerMAX.

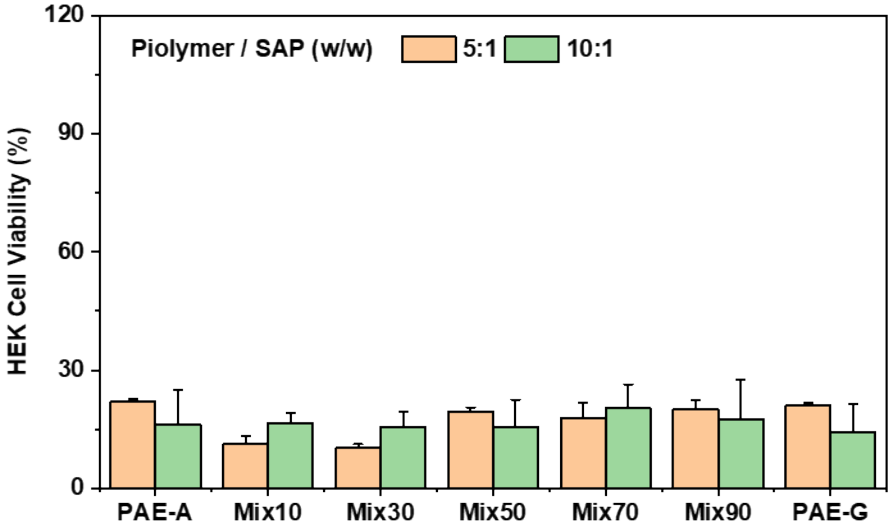

Figure S22. HEK cell viability after treatment with Saporin (SAP) delivered by different polymers at polymer/SAP weight ratios of 5:1 and 10:1.

Table S1. Quantitative coupling relationships from 2D-NMR

COSY	g+h,b	i,f	l+j,d	n+o,b,a
PAE-A	1.052	0.994	0.594	0.277
PAE-G	1.208	0.397	0.205	0
PAE-Mix50	1.103	0.245	0.340	0
**TOCSY**	**g+h,b**	**i,f+d+e**	**l+j,f+d+e**	
PAE-A	0.642	0.480	0.468	
PAE-G	0.667	0.413	0.429	
PAE-Mix50	0.633	0.427	0.427	

Table S2. Physical properties of polymers

	**PAE-A**	**PAE-G**	**PAE-Mix50**
pKa	5.72	5.45	5.53
pKb	8.28	8.55	8.47
Ph in water	8.02	6.45	6.94
Ph in NaAc	5.42	5.25	5.18
Degree of protonation in water (%)	0.50	9.04	3.79
Degree of protonation in NaAc (%)	66.61	61.18	69.37
